# Mesenchymal stem cell-derived extracellular vesicles targeting irradiated intestine exert therapeutic effects

**DOI:** 10.7150/thno.97623

**Published:** 2024-08-26

**Authors:** Ningning He, Mingxin Dong, Yuxiao Sun, Mengmeng Yang, Yan Wang, Liqing Du, Kaihua Ji, Jinhan Wang, Manman Zhang, Yeqing Gu, Xinran Lu, Yang Liu, Qin Wang, Zongjin Li, Huijuan Song, Chang Xu, Qiang Liu

**Affiliations:** 1 Tianjin Key Laboratory of Radiation Medicine and Molecular Nuclear Medicine, Institute of Radiation Medicine, State Key Laboratory of Advanced Medical Materials and Devices, Chinese Academy of Medical Science and Peking Union Medical College, Tianjin 300192, China.; 2 State Key Laboratory of Experimental Hematology, National Clinical Research Center for Blood Diseases, Haihe Laboratory of Cell Ecosystem, Institute of Hematology & Blood Diseases Hospital, Chinese Academy of Medical Sciences & Peking Union Medical College, Tianjin, 300020, China.; 3 School of Medicine, Nankai University, Tianjin, China.

**Keywords:** extracellular vesicles, mesenchymal stem cells, nanotherapeutics, radiation, intestinal injury

## Abstract

**Background:** Radiation-induced intestinal injuries are common in patients with pelvic or abdominal cancer. However, these injuries are currently not managed effectively. Mesenchymal stem cell-derived extracellular vesicles (MSC-EVs) have been extensively used in regenerative medicine. However, the results of MSC-EVs in the repair of radiation-induced intestinal damage have been unsatisfactory. We here investigated the nanotherapeutic functions of MSC-EVs in radiation-induced intestinal injury.

**Methods:** We visualized the biodistribution and trend of MSC-EVs through *in vivo* imaging. A radiation-induced intestinal injury model was constructed, and the therapeutic effect of MSC-EVs was explored through *in vivo* and *in vitro* experiments. Immunofluorescence and qRT-PCR assays were conducted to explore the underlying mechanisms.

**Results:** MSC-EVs exhibited a dose-dependent tendency to target radiation-injured intestines while providing spatiotemporal information for the early diagnosis of the injury by quantifying the amount of MSC-EVs in the injured intestines through molecular imaging. Meanwhile, MSC-EVs displayed superior nanotherapeutic functions by alleviating apoptosis, improving angiogenesis, and ameliorating the intestinal inflammatory environment. Moreover, MSC-EVs-derived miRNA-455-5p negatively regulated SOCS3 expression, and the activated downstream Stat3 signaling pathway was involved in the therapeutic efficacy of MSC-EVs in radiation-induced intestinal injuries.

**Conclusion:** MSC-EVs can dose-dependently target radiation-injured intestinal tissues, allow a spatiotemporal diagnosis in different degrees of damage to help guide personalized therapy, offer data for designing EV-based theranostic strategies for promoting recovery from radiation-induced intestinal injury, and provide cell-free treatment for radiation therapy.

## Background

Most patients with abdominal or pelvic tumors require radiotherapy, which is crucial for the management of malignant pelvic diseases. However, this treatment is mostly limited because of its toxicity to surrounding healthy tissues, such as the intestine 1-3. The intestine is among the most radiosensitive intra-abdominal organs. Abdominal irradiation (AIR) may affect intestinal integrity and function and cause acute and/or chronic gastrointestinal tract (GIT) disorders 4. Furthermore, 10%-20% of patients with abdominal cancer who receive radiotherapy develop intestinal complications several years after treatment completion. These chronic complications detrimentally affect the patient's quality of life, and in some cases, these complications can be life-threatening. Patients with radiotherapy-induced GIT disorders can only be treated symptomatically. At present, no curative treatment is available for these disorders.

Mesenchymal stem cells (MSCs) are considered a promising approach for tissue engineering and regenerative medicine, as reported by various clinical trials 5, 6. However, preclinical studies and clinical trials have found that only a small amount of implanted MSCs could engraft the inflamed tissue 7-9; however, therapeutic effects were often observed 10, 11. Increasing evidence suggests that MSC-secreted extracellular vesicles (EVs) play key roles in wound healing and tissue regeneration 12. Being a natural nanomaterial type, EVs offer several advantages, such as abundant sources, low immune rejection, and flexibility in re-engineering. They can also be used readily as cell-free therapeutics 13, 14. Moreover, EVs can modulate the immune system 15 and are being extensively tested as a nanotherapeutic agent for diabetes 16, stroke 17, 18, and wound healing 19, 20. However, to the best of our knowledge, the therapeutic effects and action mechanisms of MSC-EVs in radiation-induced intestinal injury remain poorly understood. We here assessed the significance of MSC-EV intestinal homing in resolving radiation-induced intestinal injury and investigated the mechanisms through which MSC-EVs elicit their therapeutic activities.

Signal transducers and activators of transcription 3 (Stat3), the most prominent Stat family member, are involved in several cellular processes including development, differentiation, inflammation, and metabolism by mediating cytokine signaling 21. Stat3 is typically activated by janus kinase (Jak) 2-mediated phosphorylation of its tyrosine at position 705. Following phosphorylation, Stat3 dimerizes, translocates into the nucleus, and binds to Stat3-binding elements in the regulatory regions of its target genes. Stat3 is involved in the self-renewal of mouse embryonic stem cells 22, survival of murine small intestine crypts, and intestinal homeostasis 23, 24.

MicroRNAs (miRNAs) are small noncoding RNAs (nucleotides: 21-24) functioning as critical post-transcriptional gene regulators 25. MiR-455-5p has garnered increasing research attention and is known to participate in multiple biological processes. It exerts its regulatory functions by targeting the suppressor of cytokine signaling 3 (SOCS3) 26. SOCS3 encodes a gene that can bind to JAK2 and inhibit its activity 27. Furthermore, SOCS3 is the key inhibitor of the Janus kinase/signal transducer and activator of the transcription 3 (JAK/Stat3) signaling pathway 28. However, the action mechanism of miR-455-5p/SOCS3/Stat3 in radiation-induced small intestine injury remains unclear. We here investigated the miR-455-5p/SOCS3/Stat3-mediated putative therapeutic effects of MSC-EVs on cell proliferation, apoptosis, and inflammatory responses in AIR mice.

**Scheme 1:**
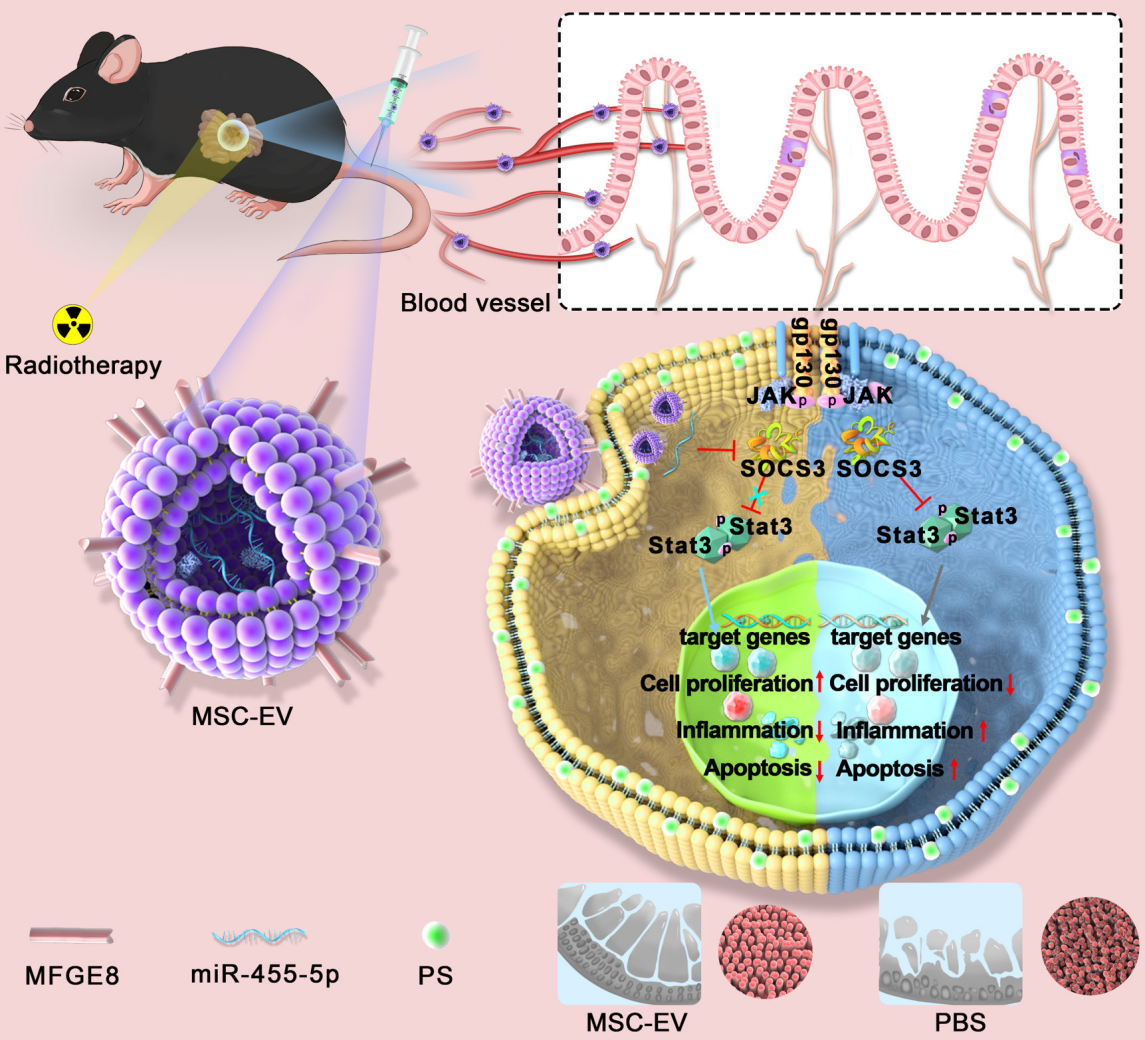
** An overview of the proposed mechanisms of MSC-EVs-mediated therapeutic effects in radiation-induced intestinal injury.** On tail vein injection, MSC-EVs could reach the intestine through blood circulation and targeted the injured intestine by binding MFGE8 to PS on the injured intestinal epithelial cells. MSC-EVs contained miR-455-5p that probably exerted their therapeutic efficacy by downregulating inflammation and apoptosis and promoting cell proliferation through the miR-455-5p/SOCS3/Stat3 pathway.

MSC-EVs injected through the tail vein actively targeted the intestine through blood circulation (**Scheme 1**). They had a complex mixture of miRNAs targeting different signaling pathways. In our study, MSC-EVs targeted the injured intestine by binding milk fat globule-epidermal growth factor 8 (MFGE8) to phosphatidylserine (PS) on the injured intestinal epithelial cells and releasing therapeutic factors present in the MSC-EVs to protect MODE-K cells or mice against radiation-induced injury. Here, we determined whether miR-455-5p-containing MSC-EVs probably exerted their therapeutic efficacy by downregulating inflammation and apoptosis and promoting cell proliferation through the miR-455-5p/SOCS3/Stat3 pathway. Our results suggested that MSC-EVs can serve as a nanotherapeutic agent for repairing radiation-induced intestinal injury.

## Methods

### Cells

Human placenta-derived MSCs were cultured, as reported 29. To collect MSC-EVs, 80% of confluent MSCs were cultured in complete media supplemented with 10% EV-depleted FBS for 2 days. The murine small intestinal epithelial cell line MODE-K was cultured in RPMI-1640 containing 10% FBS, 100 U/mL penicillin, and 100 μg/mL streptomycin.

### MSC-EV characterization

MSC-EVs were analyzed through transmission electron microscopy (TEM) following our published protocol 30. In the nanoparticle tracking analysis, EVs (1 μg/μL) were diluted 1:4000 with distilled water and injected into the channel to analyze the EV size and concentration. The size distributions and particle concentrations of EVs were analyzed using a minimum area of 10, a maximum area of 1000, a minimum brightness of 30, and a camera at 0.703 µm/px, and a temperature of 25 °C. EV marker proteins (CD63, CD81, CD9, TSG101, and the negative maker Calnexin) were analyzed through western blotting.

### *In vitro* tracking of EVs through PKH26 staining

To track EV uptake *in vitro*, the EVs were stained with the PKH26 (Sigma, MKCJ8712) fluorescent linker 31. Briefly, 2 μL PKH26 was diluted in 500 μL diluent C (A tube), and 30 μg EVs were diluted in 500 μL PBS (B tube). Contents of the A and B tubes were mixed continuously for 30 s by gentle pipetting. Then, the mixture was incubated for 5 min at room temperature. Dyeing was stopped by adding an equal volume (1 mL) of 1% EV-depleted FBS. The mixture was ultracentrifuged at 100,000 *g* for 2 h, and the EV pellet was resuspended in 1× PBS through gentle pipetting.

### *In vivo* tracking of EVs through near-infrared imaging

C57BL/6J male mice (age: 6-8 weeks) were used for the *in vivo* experiment. According to the different groups, the mice were exposed to abdominal radiation doses of 2, 8, and 15 Gy to induce different severities of radiation injury. EVs were labeled with the ExoGlow-Vivo near-infrared (NIR) dye (SBI) at a concentration of 2 μL NIR dye in a maimum of 250 μg protein equivalent of EVs and intravenously injected into the mice to visualize MSC-EV biodistribution and trend by using the IVIS 200 small animal imaging system (PerkinElmer, Waltham, MA, USA). At the indicated time points, the mice were immediately imaged, and the EVs were tracked by NIR signals. At the end of the experiments (6 or 24 h after EV injection), the mice were sacrificed, and their tissues (lungs, heart, liver, spleen, kidneys, and intestines) were dissected and imaged immediately. The average fluorescence of each tissue sample was obtained. The images were acquired and analyzed using Living Image software (PerkinElmer).

### Isolation of miR-455-5p-diminished EVs

MSCs were grown to 80% confluence and transfected with 100 nM oligonucleotide (RiboBio, Guangzhou, China) by using Lipofectamine 2000 in Opti-MEM (Invitrogen). Nontargeting control (NC) RNAs were used as controls. After the cells were transfected for 4 h, the cells were maintained in DMEM/F12 medium containing EV-depleted FBS for an additional 48 h. The conditioned media were collected, and the miR-455-5p-diminished EVs were harvested.

### Cell proliferation

MODE-K cells (5×10^4^) were seeded in 12-well plates. The medium was replaced with 100 μg/mL of MSC-EVs and mixed with RPMI-1640 (containing 10% EV-depleted FBS) on the next day. Cells in the plate were counted in three days.

### Irradiation

Cells or animals were placed in the center of an irradiation chamber and exposed to a radiation dose of 1 Gy/min emitted from a Gammacell-40 ^137^ Cesium gray irradiator (Atomic Energy Co., Atomic Energy of Canadian Inc, Mississauga, ON, Canada).

### Intestinal injury mode and EV transplantation

All animal breeding and experiments were conducted following the protocols of the Animal Experiment Ethics Committee and Authority of the Institute of Radiation Medicine, Chinese Academy of Medical Sciences [Approval No: SYXK (Jin) 2019-0002]. First, 6-to-8-week-old male C57BL/6J mice were housed under standard laboratory conditions. The animals were exposed to an AIR dose of 15 Gy to induce intestinal radiation injury. Non-irradiated mice were used as the control group, and the irradiated mice were randomized into the PBS and MSC-EV groups (n = 5 per group). The MSC-EV group was intravenously injected with 100 μg EVs on days 0, 2, and 3 after irradiation. The mice were euthanized on days 4 and 14 after irradiation to examine radiation-induced intestinal injury. On day 4, endoscopy was performed after the mice were euthanized. The progression and severity of intestinal injury were assessed on the basis of macroscopic damage. Intestinal damage was evaluated and scored in a blinded manner 32, 33 according to the following criteria: 0: normal appearance, 1: focal hyperemia, without ulcers, 2: ulceration without hyperemia or bowel wall thickening, 3: ulceration with inflammation at one site, 4: ulceration or inflammation at two or more sites, 5: major sites of damage extending 1 cm along the colon length, and 6-10: area of damage extending 2 cm along the colon length. The score was increased by 1 for each additional centimeter of involvement.

### Histology analysis

For the histology analysis, the animals were euthanized at the indicated time points. Their intestinal tissues were isolated, fixed in 4% paraformaldehyde for 24 h, dehydrated with gradient ethanol, hyalinized with xylene, embedded in paraffin, and cut into 5-μm paraffin sections. To obtain frozen sections, the samples were washed with PBS, cryoprotected in 30% sucrose in PBS, embedded in OCT (Sakura Finetek, Japan), and transversely cut using a cryostat (Leica Biosystems Nussloch GmbH, Germany) to produce 5-μm-thick sections. The paraffin sections were stained with hematoxylin and eosin according to the standard protocol. For immunohistochemical staining, the paraffin sections were antigen-repaired and incubated with a primary antibody against lysozyme (ab108508, Abcam). In immunofluorescence staining, the cryosections were first incubated with a primary antibody against Ki67 (ab15580, Abcam) or γH2AX (Ser139) (ab81299, Abcam).

### Real-Time PCR

Total RNA was extracted from the cells or mouse intestine tissue using Trizol (Invitrogen). The extracted RNA was reverse transcribed into cDNA by using a reagent kit (RR047B and 638315, Takara, China). Real-time PCR was performed using the SYBR Green reagent (CW0957W, CWBIO, China) on the Real-time PCR System (CFX connect, Bio-Rad, USA). Using the 2-ΔΔCt method, the relative gene and miRNA expression was determined after it was normalized to that of the endogenous housekeeping gene GAPDH and U6. Primer sequences are presented in Table S1 of Additional File 1.

### Western blotting

EV pellets, cells, and intestine tissues were lysed with RIPA lysis buffer (Solarbio, Shanghai, China). The protein concentration was measured using a BCA protein assay kit (Promega). The procedures were performed as previously described 34. The following primary antibodies were used: CD63 (A19023, Abclonal), CD81 (52892S, CST), CD9 (13174S, CST), Calnexin (2679S, CST), TSG101 (ab125011, Abcam), pSTAT3 (Y705) (ab76315, Abcam), SOCS3 (ab16030, Abcam), VEGFR1 (ab32152, Abcam), Survivin (ab134170, Abcam), β-tubulin (Proteintech, 66240-1-Ig), β-actin (Proteintech, 60008-1-Ig), and GAPDH (Proteintech, 60004-1-Ig).

### Enzyme-linked immunosorbent assay

The levels of IL-1β, IL-6, IL-10, TNF-α, and IL-22 in the cell supernatant and serum from experimental mice were detected as per the manufacturer's instructions (R&D).

### Statistical analyses

All statistical analyses were performed using GraphPad Prism software. Data were expressed as the mean ± SD. The two-tailed Student's-test was employed for comparisons between the two groups, while one-way ANOVA was performed for comparison of data among more than two groups. *P* < 0.05 was considered to indicate statistical significance.

## Results

### MSC-EV characterization

**Figure 1:**
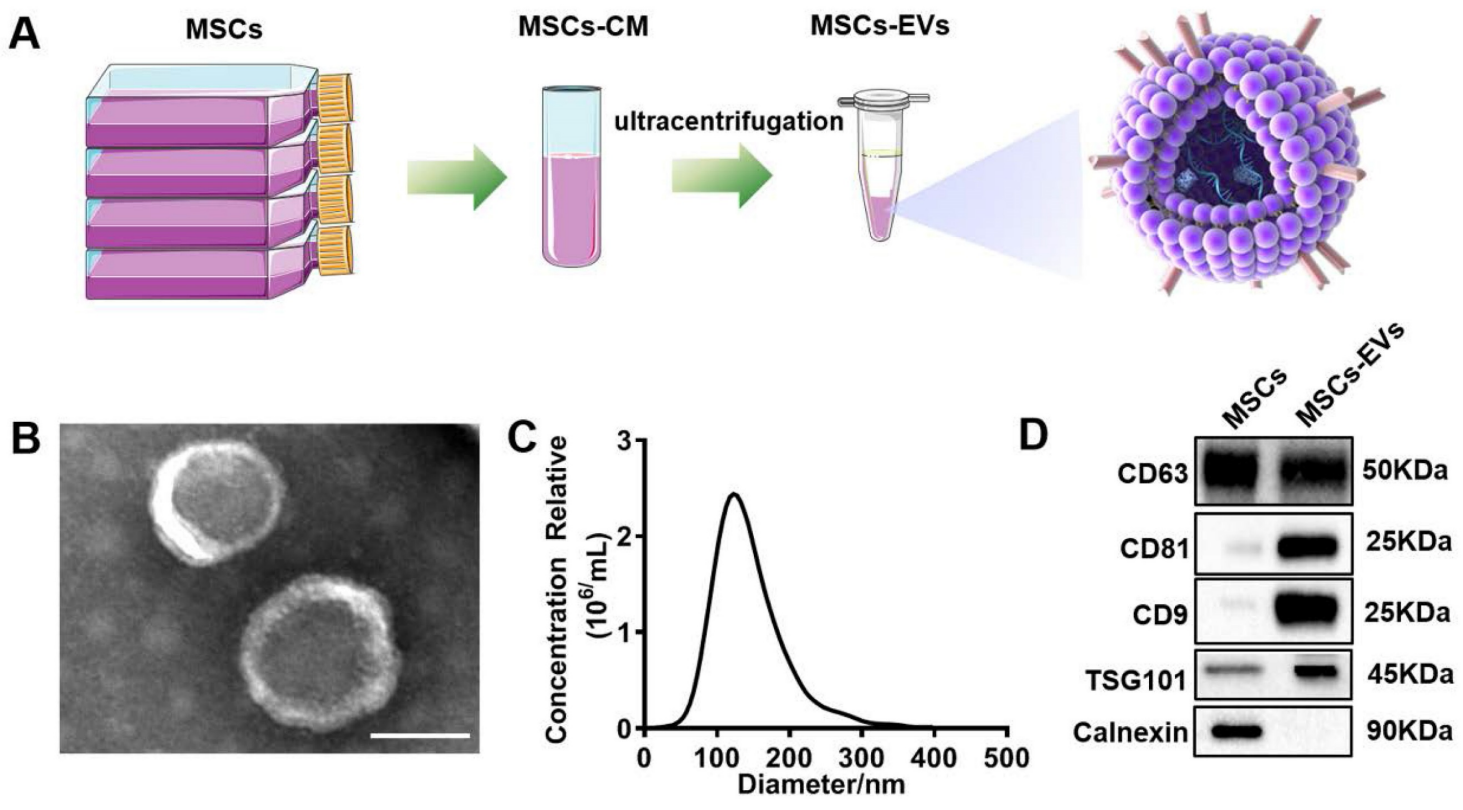
** Characterization of extracellular vesicles (EVs).** (A) Extraction of EVs from MSCs supernatants. (B) Transmission electron microscopy (TEM) image of EVs derived from MSCs. Scale bars = 100 nm. (C) NTA analysis of the diameter of EVs. (D) Western blotting of the EVs markers CD63, CD81, CD9, TSG101, Calnexin, and their parent cells (MSCs).

MSC-EVs were isolated from the MSC-conditioned medium through sequential ultracentrifugation (Figure 1A). The isolated MSC-EVs were characterized using different methods, including TEM, NTA, and western blotting. Spherical vesicles with clear membrane structures within the EV size range were observed through TEM (Figure 1B). The NTA determined that the average EV size was approximately 130 nm in diameter (Figure 1C). Western blotting unveiled the EV markers CD63, CD81, CD9, and TSG101 and the negative maker Calnexin (Figure 1D).

### MSC-EVs exhibited an ability to preferentially target irradiated intestines and could be used for monitoring the severity of radiation-induced intestinal injury

**Figure 2:**
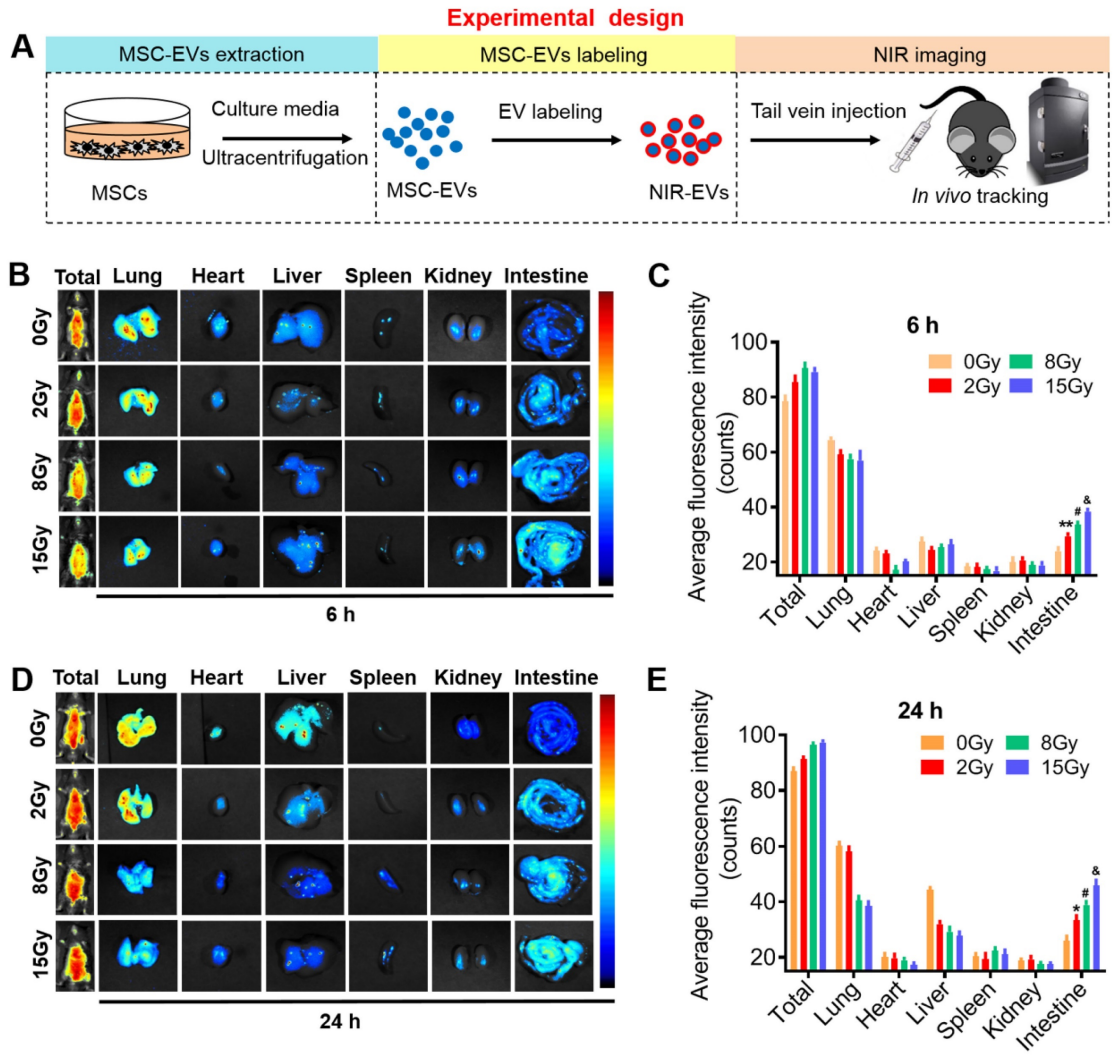
** Real-time imaging of MSC-EVs in radiation-induced intestinal injury mice.** (A) *In vivo* tracking in the EV experiment illustration. (B and D) Organ distributions of EVs at the designed time points after NIR-EVs administration. Injury induced by 15-Gy abdominal radiation enhanced the intestine retention and stability of EVs compared to that in the 0-Gy group. (C and E) Quantitative analysis of the fluorescence intensity of EVs in the organs of mice. ^*^P < 0.05 *vs.* 0 Gy, ^**^P < 0.01 *vs.* 0 Gy, ^#^*P* < 0.05 *vs.* 2 Gy, ^&^*P* < 0.05 *vs.* 8 Gy, n = 3.

To investigate whether MSC-EVs can be used as a therapeutic tool, the biodistribution and targeting mechanisms of MSC-EVs *in vivo* and *in vitro* must be assessed. MSC-EVs exert therapeutic effects in many diseases 35-37. EVs transferring bioactive cargo to both adjacent and distant sites can influence the functions of cells or tissues located far away from the body sites where they are secreted 38, 39. To visualize EVs *in vivo*, MSC-EVs isolated through ultracentrifugation were labeled with the ExoGlow-*Vivo* NIR dye and intravenously injected into mice to visualize MSC-EVs biodistribution and trend using the *in vivo* imaging system (Figure 2A). We evaluated the retention time and distribution of labeled MSC-EVs in mice in the non-irradiation and irradiation groups. Mice were exposed to 2, 8, and 15-Gy radiation to induce different severities of intestinal injury, followed by an injection of 100 μg NIR-labeled MSC-EVs via the tail vein 24 h after radiation. At the 6th and 24th h after MSC-EV injection, the mice were imaged and sacrificed. Their organs were harvested, and the fluorescence intensity within the organ tissues was analyzed. No significant difference was detected in the fluorescence intensity of the whole mice body among the non-irradiation and the 2-, 8-, and 15-Gy irradiation groups and there were no obvious difference between 6 and 24 h of the organs fluorescence intensity. However, EV accumulation increased in the intestines after irradiation, especially from the 8- and 15-Gy irradiation groups compared with those from the non-irradiation group (Figure 2B-E). There showed no obvious dose-dependency in the intestine at 24 hours when used the NIR dye only (Figure S1). And we also examined the distribution of NIR-labeled MSC-EVs at 48 and 72 hours after injection. The results indicated that radiation injury prolongs the retention time of MSC-EVs in the irradiated intestine (Figure S2).

To visualize EVs *in vitro*, MSC-EVs were labeled with the lipophilic dye PKH26 and cocultured with the MODE-K cells irradiated at different doses. Irradiation efficiently increased the binding of MSC-EVs to the irradiated cells, which was indicated by the positive red (PKH26) signals for the colocalized cells. The green fluorescence signals indicated a phalloidin-stained cytoskeleton within these cells (Figure S3). Altogether, these results demonstrated that MSC-EVs preferentially targeted injured intestines and cells in a radiation dose-dependent manner. The severity of intestinal injury at early stage can be determined by quantifying the amount of MSC-EVs (the signal intensity) accumulated in the injured tissue. Thus, MSC-EVs are a promising candidate for the early diagnosis of radiation injury.

### MFGE8 mediated MSC-EVs binding to PS on the irradiated MODE-K cells

**Figure 3:**
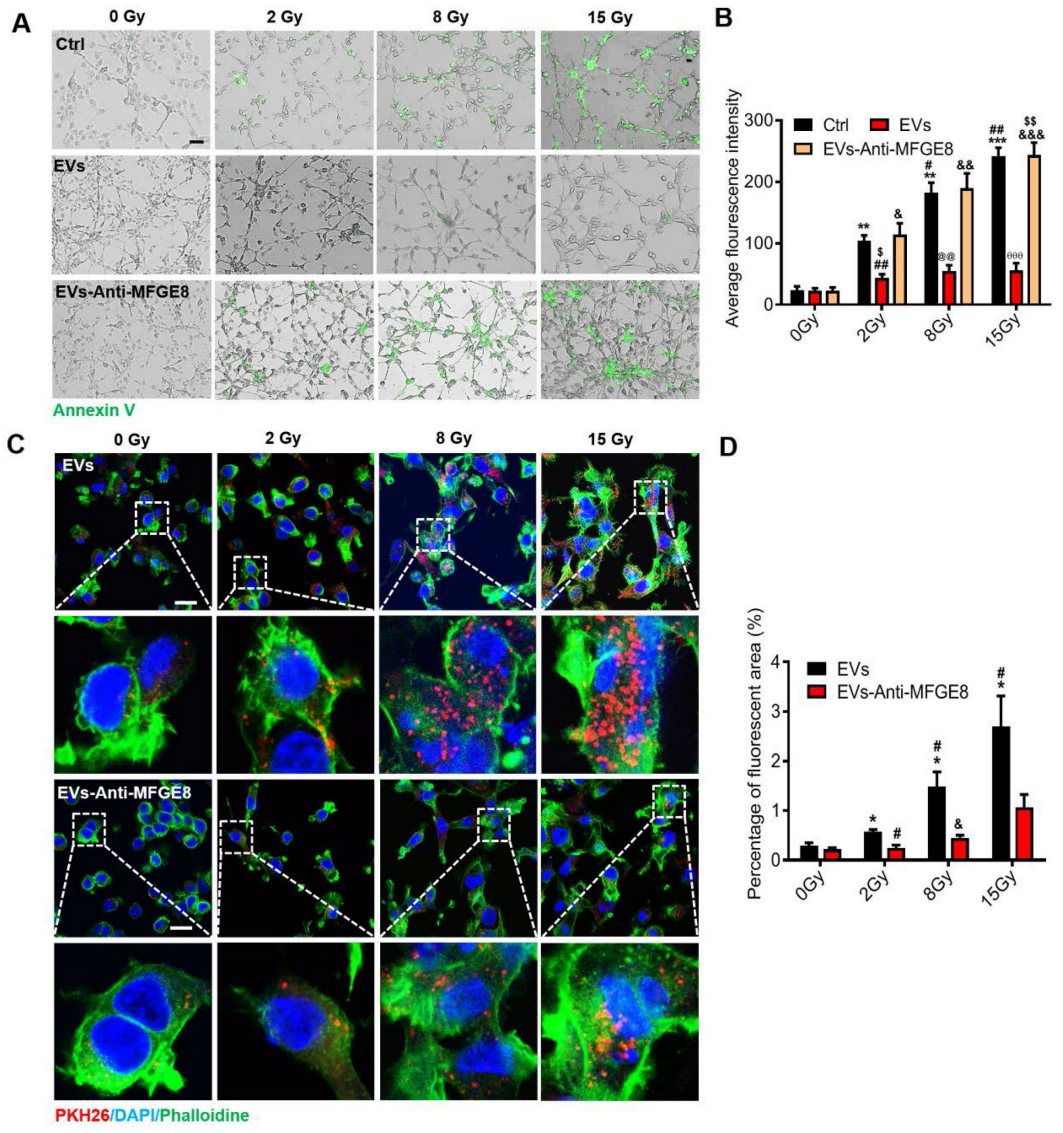
** MSC-EVs target irradiated cells in a radiation dose-dependent manner via the MGFE8 on the MSC-EVs.** (A) Representative immunofluorescence staining image showing annexin V fluorescein isothiocyanate (green)-stained apoptotic MODE-K cells treated with different conditions under different irradiation doses. Scale bar = 100 μm. (B) Graph depicting that irradiation increases the positive proportion of early apoptosis cells represented as the average fluorescence intensity. **P < 0.01 *vs.* 0 Gy-Ctrl, ***P < 0.001 *vs.* 0 Gy-Ctrl, ^#^*P* < 0.05 *vs.* 2 Gy-Ctrl, ^##^*P* < 0.01 *vs.* 2 Gy-Ctrl , ^&^*P* < 0.05 *vs.* 0 Gy-EVs-Anti-MFGE8, ^&&^*P* < 0.01 *vs.* 0 Gy-EVs-Anti-MFGE8,^ &&&^*P* < 0.001 *vs.* 0 Gy-EVs-Anti-MFGE8, ^$^*P* < 0.05 *vs.* 2 Gy-EVs-Anti-MFGE8, ^@@^*P* < 0.01 *vs.* 8 Gy-Ctrl and 8 Gy-EVs-Anti-MFGE8, ^θθ^*P* < 0.01 *vs.* 15 Gy-Ctrl and 15 Gy-EVs-Anti-MFGE8. (C) Immunofluorescence staining showing the uptake of PKH26-labeled MSC-EVs or the use of MFGE8-neutralizing antibody pretreated MSC-EVs (red) by MODE-K cells. The inset represents a magnified view of the boxed area. Scale bar = 25 μm. (D) Graph depicting the percentage uptake of fluorescent MSC-EVs or using MFGE8 neutralizing antibody-pretreated MSC-EVs (red) by MODE-K cells. The results show MGFE8 plays a role in increasing the accumulation of MSC-EVs into irradiated cells. *P < 0.05 *vs.* 0 Gy-EVs, ^#^*P* < 0.05 *vs.* 2 Gy-EVs, ^&^*P* < 0.05 *vs.* 8 Gy-EVs, n = 3.

Encouraged by the aforementioned findings, we explored the mechanism underlying the targeting ability of MSC-EVs in the MODE-K cells. Staining with annexin V fluorescein isothiocyanate, a phospholipid-binding protein with a high affinity for PS, revealed that the amount of PS on the surface of the irradiated MODE-K cell membranes increased after exposure to 2-, 8, and 15-Gy irradiation. Interestingly, the amount of PS on the surface of the irradiated cell membranes depended on the irradiation dose (Figure 3A). The membrane-associated protein MFGE8 is enriched in MSC-EVs and is located on the outside of the EVs 40. MFGE8 can bind to PS on apoptotic cells 3. We hypothesized that MSC-EVs are targeted to irradiated cells through the binding of MFGE8 to the PS on the cell membrane. To verify this, an MFGE8-neutralizing antibody was used. The MODE-K cells were incubated for 6 h at 37 °C after 2-, 8-, and 15-Gy irradiation. MSC-EVs or MSC-EVs pretreated with the MFGE8-neutralizing antibody were added to the MODE-K cell culture medium. After incubation for 2 h, the cells were washed with PBS, and annexin V fluorescein isothiocyanate was added. The cells were incubated with the stain for 30 min to detect the fluorescence intensity. The annexin V fluorescence intensity of the cells significantly decreased after MSC-EV treatment. This illustrated that MSC-EVs might bind to the irradiated cells preventing binding of annexin V to the MODE-K cells. Nevertheless, the MFGE8-neutralizing antibody significantly inhibited the binding of MSC-EVs to the MODE-K cells, which in turn increased the binding of annexin V to the cells (Figure 3A, B).

Fluorescent images of PKH26-labeled MSC-EVs in the MODE-K cells revealed the same results. This suggested that more MSC-EVs can be internalized by the irradiated MODE-K cells when MFGE8 binds to PS on these cells. Subsequently, an MFGE8-neutralizing antibody distinctively suppressed MSC-EVs internalization by the irradiated cells (Figure 3C, D).

### Preventive effects of MSC-EVs on intestinal epithelial cells

**Figure 4:**
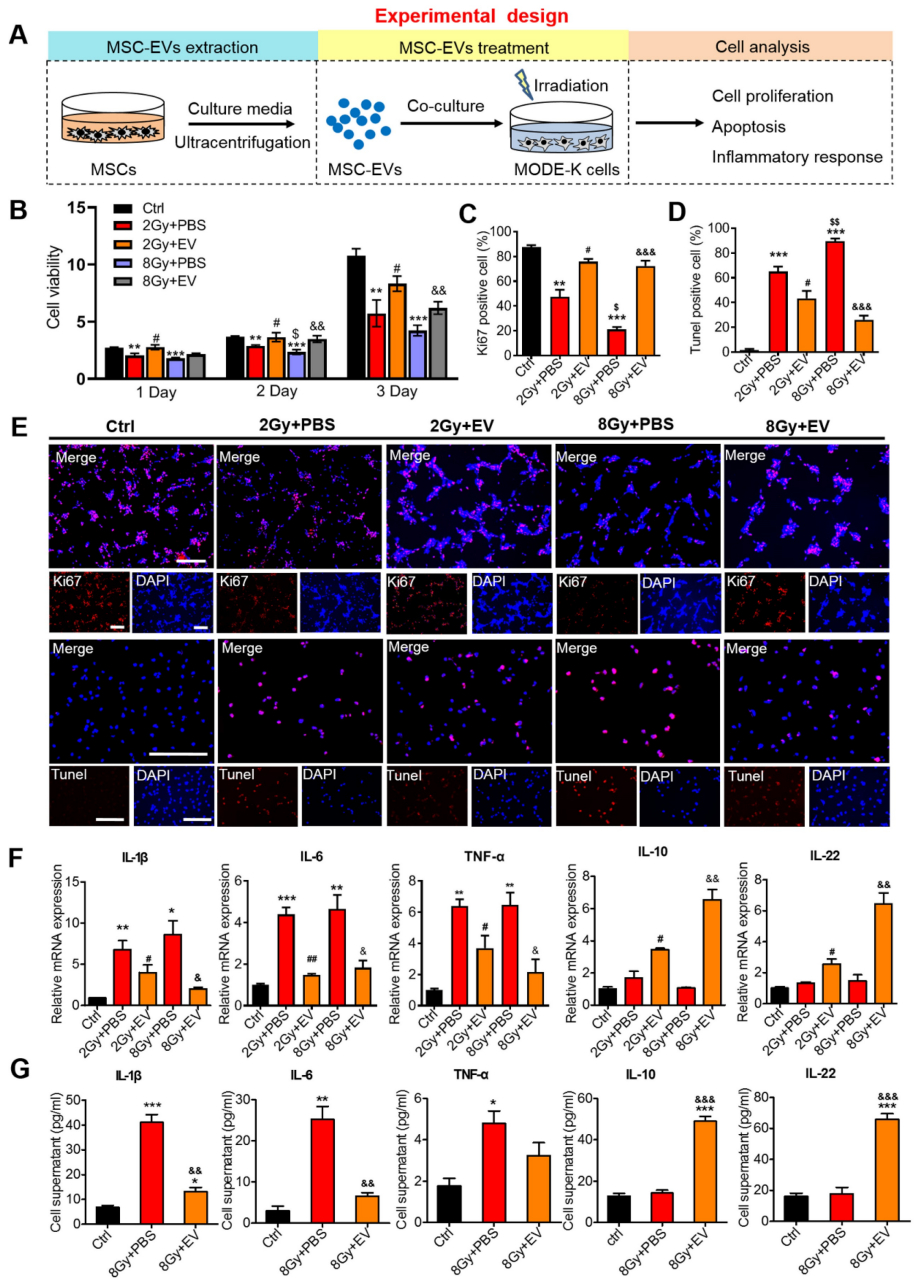
** Preventive effects of MSC-EVs on MODE-K cells.** (A) An *in vitro* experiment illustration. (B) The cell viability increased in MSC-EVs treated groups. ^**^P < 0.01 *vs.* Ctrl, ^***^P < 0.001 *vs.* Ctrl, ^#^*P* < 0.05 *vs.* 2 Gy+PBS, ^$^*P* < 0.05 *vs.* 2 Gy+PBS, ^&&^*P* < 0.01 *vs.* 8 Gy+PBS, n = 3. (C, D and E) Quantitative data and representative images of cell proliferation and apoptosis. MSC-EVs attenuated apoptosis and promoted the proliferation of MODE-K cells after radiation. Scale bar = 100 μm. Nuclei were counterstained with DAPI (blue). ^**^P < 0.01 *vs.* Ctrl, ^***^P < 0.001 *vs.* Ctrl, ^#^*P* < 0.05 *vs.* 2 Gy+PBS, ^$^*P* < 0.05 *vs.* 2 Gy+PBS, ^$$^*P* < 0.01 *vs.* 2 Gy+PBS, ^&&&^*P* < 0.001 *vs.* 8 Gy+PBS, n = 3. (F) qRT-PCR analysis revealed the expression of inflammation-related genes. The EV treatment decreased the expression of pro-inflammatory genes (IL-1β, IL-6, and TNF-α) and increased the expression of anti-inflammatory genes (IL-10 and IL-22). ^**^P < 0.01 *vs.* Ctrl, ^***^P < 0.001 *vs.* Ctrl, ^#^*P* < 0.05 *vs.* 2 Gy+PBS, ^##^*P* < 0.01 *vs.* 2 Gy+PBS, ^&^*P* < 0.05 *vs.* 8 Gy+PBS, ^&&^*P* < 0.01 *vs.* 8 Gy+PBS, n = 3. (H) ELISA analysis of inflammatory cytokines of the MODE-K cell supernatants. The EV treatment decreased the expression of pro-inflammatory cytokines (IL-1β, IL-6, and TNF-α) and increased the expression of anti-inflammatory cytokines (IL-10 and IL-22). ^*^P < 0.05 *vs.* Ctrl, ^**^P < 0.01 *vs.* Ctrl, ^***^P < 0.001 *vs.* Ctrl, ^&&^*P* < 0.01 *vs.* 8 Gy+PBS,^ &&&^*P* < 0.001 *vs.* 8 Gy+PBS, n = 3.

To determine the therapeutic effects of MSC-EVs on the repair of radiation-induced damage to intestinal epithelial cells (MODE-K), cell proliferation, apoptosis, and inflammation-related factors were assessed (Figure 4A). Compared with the irradiation group (2 Gy+PBS or 8 Gy+PBS), the cell viability increased in the MSC-EVs groups (2 Gy+EV and 8 Gy+EV) (Figure 4B). Staining with the proliferation marker Ki67 unveiled that similar dynamic changes occurred in Ki67^+^ cells in both groups (Figure 4C, E). The MSC-EV treatment effectively promoted MODE-K cell proliferation after irradiation regardless of the radiation dose (2 or 8 Gy). The number of terminal deoxynucleotidyl transferase (dUTP) nick end labeling (TUNEL)-positive nuclei indicates the severity of apoptosis. Compared with the control group, irradiation increased the number of TUNEL-positive cells in the apoptotic cells in the 2- and 8-Gy groups. MSC-EV treatment evidently decreased the number of TUNEL-positive cells after radiation (Figure 4D, E). Taken together, these results indicated that MSC-EVs attenuated apoptosis and promoted MODE-K cell proliferation following irradiation.

To evaluate the effect of MSC-EVs on the inflammatory response following radiation-induced intestinal injury, the expression of interleukin-1β (IL-1β), interleukin-6 (IL-6), tumor necrosis factor-α (TNF-α), interleukin-10 (IL-10), and interleukin-22 (IL-22) was evaluated in MODE-K cells. Following irradiation, the levels of several pro-inflammatory cytokines, such as IL-1β, IL-6, and TNF-α, were upregulated, thus mediating the inflammatory response 41. In our irradiation groups, IL-1β, IL-6, and TNF-α mRNA expression levels increased, whereas the MSC-EV treatment significantly reversed these increases (Figure 4F). Anti-inflammatory cytokines, such as IL-10 and IL-22, are protective and therapeutic players in intestinal injury. These cytokines induce the expression of antiapoptotic and proliferation-promoting proteins by activating the STAT3 signaling pathway 42. The MSC-EV treatment significantly increased the IL-10 and IL-22 levels (Figure 4F). To further investigate the immunomodulatory effect of MSC-EVs, the enzyme-linked immunosorbent assay (ELISA) was conducted to assess the levels of inflammatory factor proteins. The levels of anti-inflammatory cytokines were upregulated after irradiation, whereas the levels of pro-inflammatory cytokines were decreased by the MSC-EV treatment following irradiation (Figure 4G). This suggested that MSC-EVs induced downregulation of the pro-inflammatory response after irradiation.

### Effects of MSC-EVs on DNA damage responses

The most severe type of ionizing radiation-induced DNA damage is DNA double-strand breaks (DSBs) 43. Histone H2AX phosphorylation to γH2AX is a hallmark of DSB 44. Therefore, we evaluated DSBs through immunofluorescence staining to analyze γH2AX and thus determined the effect of MSC-EVs on DNA damage in the MODE-K cells. According to our results, γH2AX expression increased in the irradiation groups compared with the control group, and MSC-EV treatment ameliorated the damage (Figure S4).

### MSC-EVs ameliorated radiation-induced intestinal damage

**Figure 5:**
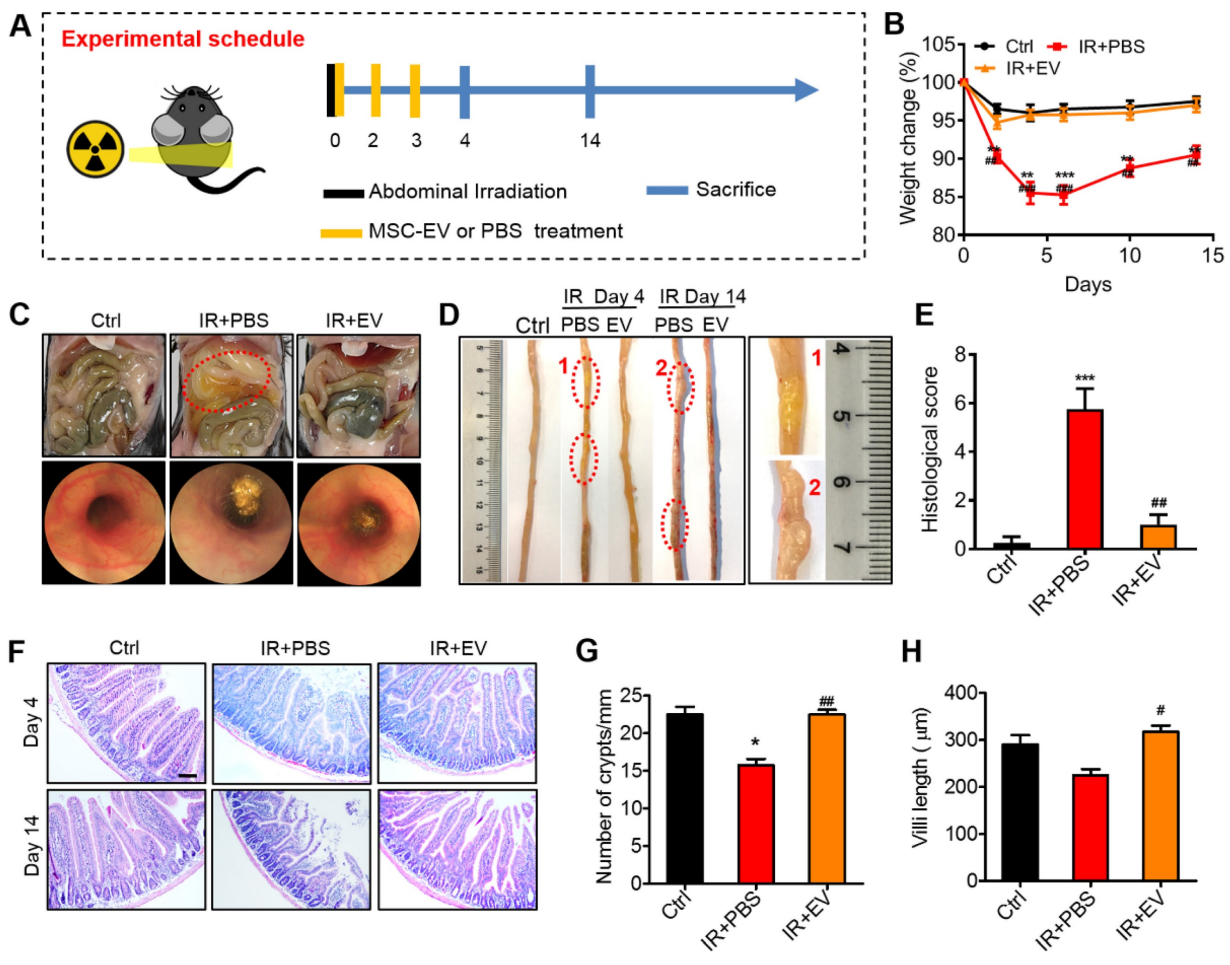
** Preventive effects of MSC-EVs on mice intestine.** (A) *In vivo* radiation experiment illustration. (B) The effect of MSC-EVs on the body weight change, MSC-EVs treatment reduced body weight loss in the irradiated mice. ^**^P < 0.01 *vs.* Ctrl, ^***^P < 0.001 *vs.* Ctrl, ^##^*P* < 0.01 *vs.* IR+PBS, ^###^*P* < 0.001 *vs.* IR+PBS, n = 4. (C) Representative macroscopical mice intestines and endoscopic images of the intestines after 4 days of radiation. The tissues in the red circle display inflammatory cell infiltration in the PBS group. The endoscopic image of mice in the PBS group showed a thickened submucosa after radiation. (D) Representative images of the intestinal tissues of mice treated with EVs or PBS after exposure to abdominal radiation. The tissues in the red circle show edema and inflammatory cell infiltration in the PBS group. (E) Histological score of intestinal inflammation. The histological score depicts a statistically significant increase in PBS when compared to that in the control group. The administration of EVs significantly reduced intestinal damage. ^***^P < 0.001 *vs.* Ctrl, ^##^*P* < 0.01 *vs.* IR+PBS, n = 4. (F) Representative micrographs for H&E staining of intestine sections on days 4 and 14. The administration of MSC-EVs largely prevented histopathological alterations after radiation injury. Scale bar = 100 μm. (G) Crypts in a single field of intestines view after 4 days of radiation were quantified. ^*^P < 0.05 *vs.* Ctrl, ^##^*P* < 0.01 *vs.* IR+PBS. (H) The villi length in a single field of intestines view after 4 days of radiation was quantified. ^#^*P* < 0.05 *vs.* IR+PBS, n = 4.

To identify the therapeutic effects of MSC-EVs, we first established a radiation-induced intestinal injury mouse model by using 15-Gy AIR 45. After receiving irradiation at 0 days, MSC-EVs were injected into the mice in the treatment group via the tail vein. The mice were then treated with MSC-EVs twice on the 2nd and 3rd day. To investigate the repair of intestinal injury, tissue samples were collected on the 4th and 14th day after irradiation (Figure 5A). The effect of MSC-EVs on the body weight of C57BL/6J mice was tested. The mice in the irradiation group exhibited a body weight loss after irradiation. However, the MSC-EV treatment significantly reduced the body weight loss in the irradiated mice (Figure 5B). Endoscopy and histologic analysis revealed that the irradiated mice developed massive erosive lesions in the mucosa and had a thickened submucosa, whereas the EV-treated mice displayed very few erosive lesions, a more intact mucosa, and a thinner submucosal layer (Figure 5C, E). Edema and inflammatory cell infiltration in the mouse intestinal tissues in the PBS group compared with the MSC-EV treatment groups (a red circle in Figure 5D). These phenomena were confirmed through H&E staining. In the irradiation group, histology revealed pathological changes in the mice, including mucosal injury, tissue structure loss, edema, and inflammatory cell infiltration, compared with the control group. However, the MSC-EV treatment ameliorated these phenomena (Figure 5F). The number of crypts in the small intestines is possibly responsible for intestinal epithelial recovery 46. The mice in the MSC-EV treatment group had a normal crypt-villus structure along with significantly increased crypts and preserved villous length compared with the mice in the irradiation group (Figure 5G, H). The number of crypts in the irradiation group reduced, as some crypts disappeared because of the death of stem cells. By contrast, the crypt-villus architecture of the intestine in the MSC-EV-treated mice was well preserved. These results indicated that MSC-EVs ameliorated the irradiation-induced intestinal damage. Moreover, in order to verify the safety of MSC-EVs, we analyzed the heart, liver, spleen, lung, and kidney tissues of the mice in the control and MSC-EVs-administered group through H&E staining (Figure S5).

### Therapeutic effects of MSC-EVs on AIR mice

**Figure 6:**
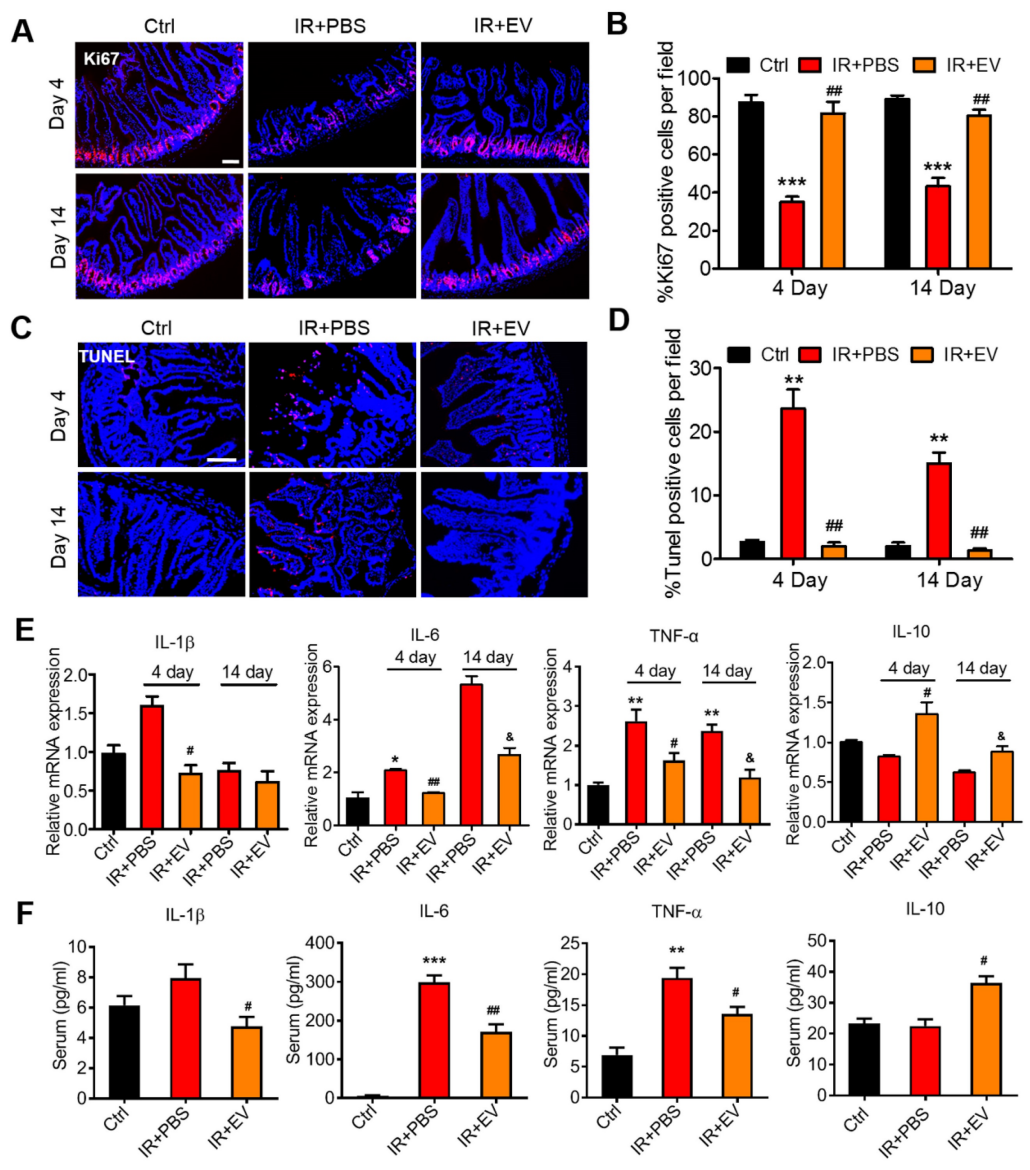
** MSC-EVs improved the viability and reduced radiation-induced apoptosis and inflammation of the intestine *in vivo*.** (A and B) Representative images of cell proliferation (Ki67, red) and quantitative data. MSC-EVs activated the proliferation of the intestine. Scale bar = 100 μm. (C and D) Representative images of cell apoptosis (TUNEL, red) and quantitative data. MSC-EVs reduced radiation-induced apoptosis in the intestine. Scale bar = 100 μm. Nuclei were counterstained with DAPI (blue) in A and C. (E) qRT-PCR analysis of inflammation-related genes. MSC-EVs reduced the inflammatory gene expression and increased the anti-inflammatory gene expression. ^*^P < 0.05 *vs.* Ctrl, ^**^P < 0.01 *vs.* Ctrl, ^#^*P* < 0.05 *vs.* IR+PBS (4 days), ^##^*P* < 0.01 *vs.* IR+PBS (4 days), ^&^*P* < 0.05 *vs.* IR+PBS (14 days), n = 3. (F) Secretion factors in the serum were analyzed by ELISA. ^**^P < 0.01 *vs.* Ctrl, ^***^P < 0.001 *vs.* Ctrl, ^#^*P* < 0.05 *vs.* IR+PBS (4 days), ^##^*P* < 0.01 *vs.* IR+PBS (4 days), n = 3.

Ki67 is a nuclear antigen that exists in proliferating cells, and it is among the most commonly used proliferative cell markers. Ki67 levels also indicate radiation-induced intestinal proliferation 2. The percentage of Ki67^+^ epithelial cells was significantly higher in the MSC-EV-treated mice compared with the untreated irradiated controls. MSC-EV treatment after irradiation activated intestinal cell proliferation (Figure 6A, B).

Lysozyme is a protein secreted by Paneth cells 47, which are located at the base of small intestinal glands. These cells contribute to the first-line of defense in the mucosal gut by secreting granules filled with antimicrobial peptides, including lysozymes 48, 49. Therefore, the number of lysozyme^+^ Paneth cells was detected to further validate the preventive effect of MSC-EVs on intestinal cells in the irradiated mice. As expected, the number of lysozyme^+^ Paneth cells significantly decreased in the irradiation group compared with the control group, and the number of these cells evidently improved in the MSC-EV group compared with the irradiation group. The levels of regeneration-associated genes (Reg3b and Reg3g) increased in the MSC-EV group (Figure S6). These data demonstrated that MSC-EVs effectively improved the proliferation of small intestine crypt cells.

To further analyze the therapeutic effect of MSC-EVs on the small intestine, the TUNEL assay was conducted to assess the apoptosis of small intestinal tissues in the mice. Numerous apoptotic cells were observed in the villi of the irradiated mice compared with the control mice. MSC-EV treatment significantly reduced apoptosis in the small intestine of the mice (Figure 6C, D). TUNEL immunohistochemical results confirmed the conclusion (Figure S7).

To measure radiation-induced intestinal inflammatory responses in the mice, an experiment was conducted to detect the mRNA and protein levels of inflammatory factors through RT-PCR and ELISA, which were conducted using the intestinal tissue and serum from the mice in each group. Intestinal and serum IL-1β, IL-6, and TNF-α levels were significantly higher in the irradiated mice than in the control mice. By contrast, MSC-EV treatment significantly reduced the levels of these proteins. The levels of IL-10, the anti-inflammatory cytokine, increased in the MSC-EV treatment group after irradiation (Figure 6E, F).

### MSC-EVs ameliorated intestinal injury via miR-455-5p/Socs3/Stat3

**Figure 7:**
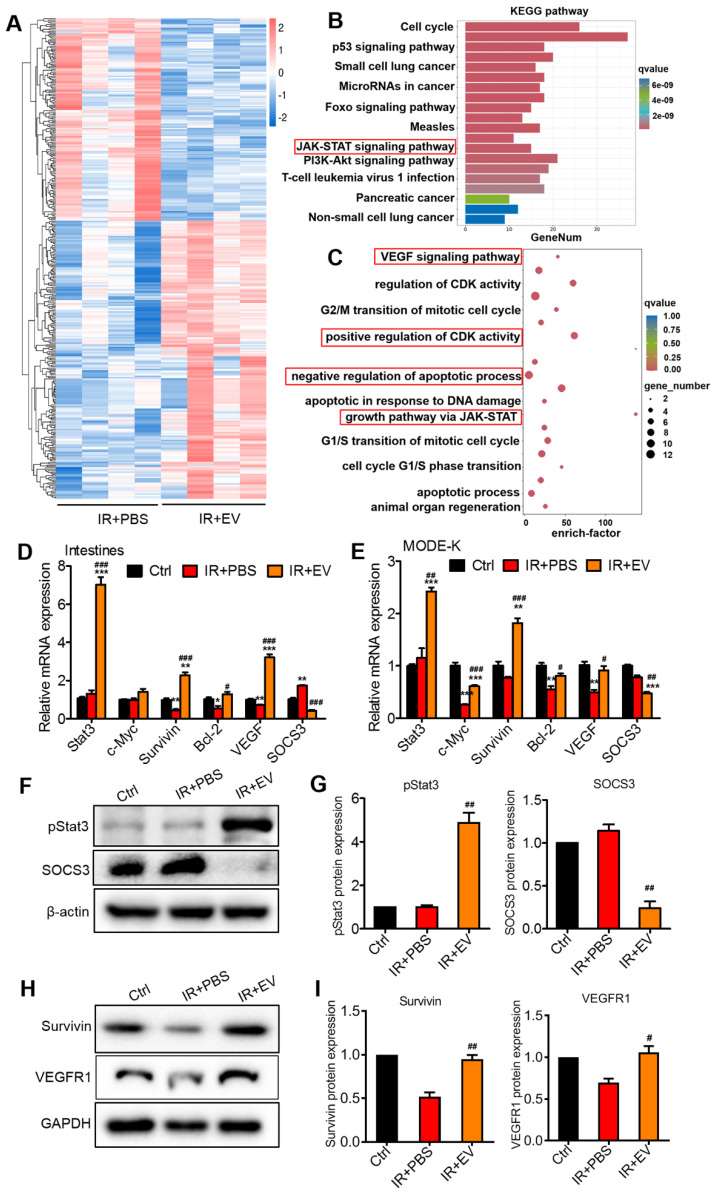
** MSC-EVs attenuated intestinal injury via miR-455-5p/SOCS3/Stat3.** (A) Hierarchical clustered heatmap displaying the differentially expressed genes. (B) The KEGG pathway analysis revealed significantly enriched signaling pathways in the differentially expressed genes. (C) GO biological processes showed differentially expressed genes in the intestine tissues after radiation treatment with EVs according to the RNA-seq data. (D) qRT-PCR analysis of the intestinal tissue showed that the genes regulated by Stat3 were upregulated after treatment with MSC-EVs. The negative regulator of Stat3, SOCS3 was reduced. ^*^P < 0.05 *vs.* Ctrl, ^**^P < 0.01 *vs.* Ctrl, ^***^P < 0.001 *vs.* Ctrl, ^#^*P* < 0.05 *vs.* IR+PBS, ^###^*P* < 0.001 *vs.* IR+PBS, n = 3. (E) qRT-PCR analysis of MODE-K cells showed that genes regulated by Stat3 were upregulated after treatment with MSC-EVs. The negative regulator of Stat3, SOCS3 was reduced. ^*^P < 0.05 *vs.* Ctrl, ^**^P < 0.01 *vs.* Ctrl, ^***^P < 0.001 *vs.* Ctrl, ^#^*P* < 0.05 *vs.* IR+PBS, ^##^*P* < 0.01 *vs.* IR+PBS, ^###^*P* < 0.001 *vs.* IR+PBS, n = 3. (F-I) Western blotting and quantitative data of Stat3-related protein expression, ^#^*P* < 0.05 *vs.* IR+PBS, ^##^*P* < 0.01 *vs.* IR+PBS, n = 3.

To explore the intercellular molecular mechanism regulated by MSC-EVs to exert the observed beneficial effects, RNA-seq was performed to compare transcriptome changes between the IR+PBS and the IR+EV groups of mouse intestine tissue following AIR. In total, 203 and 148 upregulated and downregulated genes were identified in the IR+EV group based on fold changes in expression (≥1 or ≤-1) (Figure S8). Hierarchical clustering analysis was performed on the upregulated and downregulated genes (Figure 7A). The KEGG (Kyoto Encyclopedia of Genes and Genomes) enrichment analysis was conducted to obtain information about functional annotation of differentially expressed genes. The KEGG enrichment results revealed that the differentially expressed genes in the IR+EV group were highly correlated with the Jak-Stat signaling pathway (Figure 7B), which is known to be involved in tissue repair and regeneration 50, 51. Furthermore, bioinformatics analysis was performed. According to the GO annotation classification and GO enrichment analysis, these differential genes were associated with pro-proliferation, angiogenesis, and antiapoptosis (Figure 7C). PI3-Akt and ERK pathways, which regulate cell proliferation and apoptosis, p-PI3, p-Ak and p-ERK were also detected, whereas these genes did not change between the irradiation group and the MSC-EV treatment group, so no focus was maintained on these genes in subsequent experiments (Figure S8B).

Stat3 is among the most crucial Stat family members. They play a crucial role in cell proliferation, survival, differentiation, angiogenesis, immune regulation, and other biological processes. Stat3 activation in intestinal epithelial cells triggers acute wound healing and response to injury 52. By examining Stat3 activation, we characterized the effects of MSC-EVs on intracellular signaling in the mouse intestinal tissue and MODE-K cells. After irradiation, the mice or MODE-K cells were treated with MSC-EVs. Genes regulated by Stat3, involved in cell pro-proliferation (c-Myc and Survivin), antiapoptosis (Bcl-2), and angiogenesis (VEGF), were significantly induced after the MSC-EV treatment, which was consistent with the results of the GO enrichment analysis (Figure 7D, E). The levels of SOCS3, which is known as the downstream negative regulator of Stat3, were reduced. SOCS3 plays a regulatory function in related processes 53. The p-Stat3, Survivin, and VEGFR1 protein levels increased, and the SOCS3 protein level of reduced in the MSC-EV-treated groups, which confirmed the mRNA expression results (Figure 7F-I).

**Figure 8:**
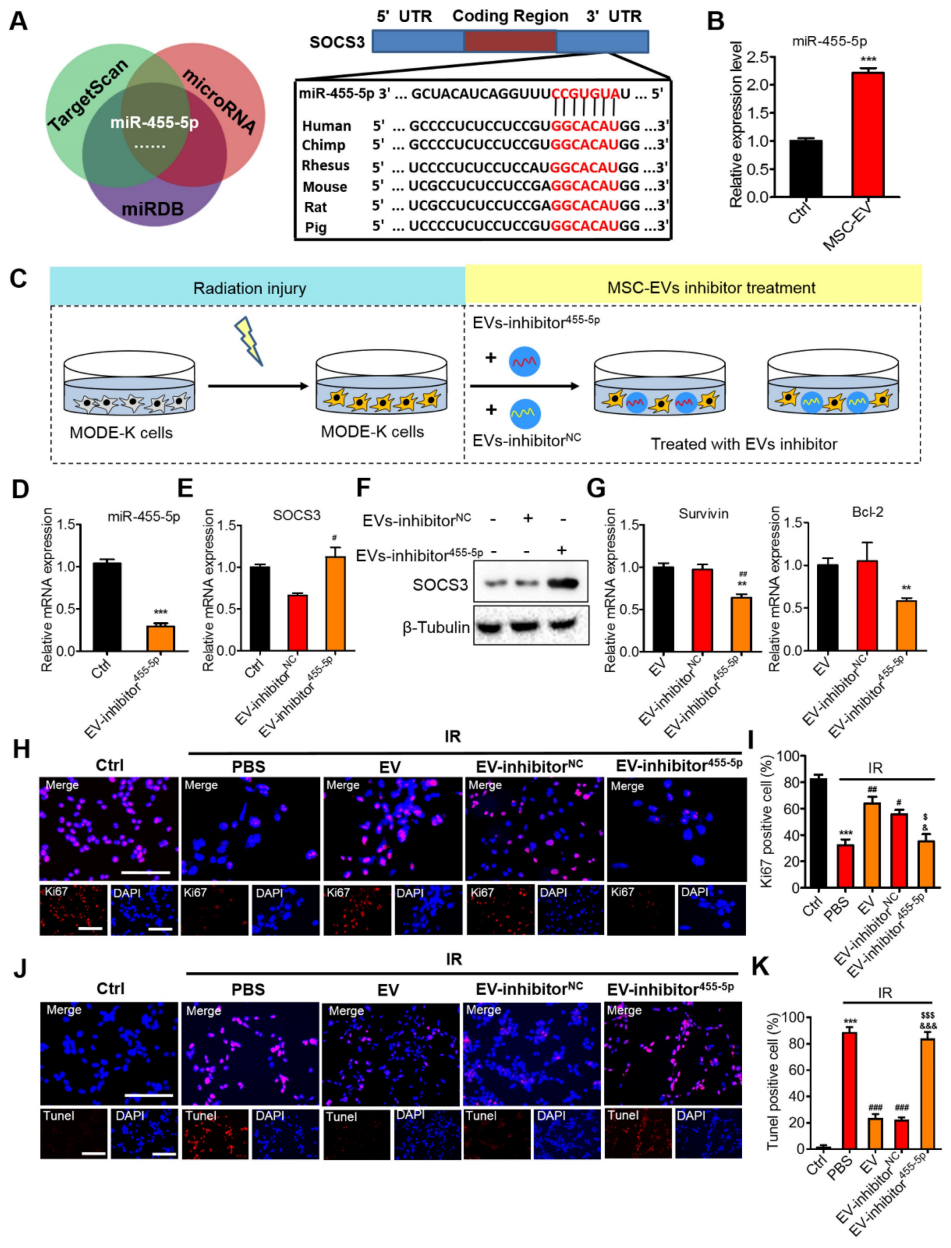
** Effects of miR-455-5p knockdown EVs on radiation-induced intestinal injury.** (A) Bioinformatic analysis of miRNAs was performed to predict the SOCS3 potential miRNA targets. MiR-455-5p was predicted to target the 3′UTR of SOCS3 mRNA in a highly conserved binding site. (B) qRT-PCR analysis revealed that the expression of miR-455-5p in MODE-K cells was increased after treatment with EVs. ^***^P < 0.001 *vs.* Ctrl, n = 3. (C) An EV inhibition experiment illustration. (D) qRT-PCR of the miR-455-5p expression in EVs after treatment with EV-miR-455-5p inhibitor. ^***^P < 0.001 *vs.* Ctrl, n = 3. (E) qRT-PCR of the target gene SOCS3 in MODE-K cells after treatment with EV-miR-455-5p inhibitor. ^#^*P* < 0.05 *vs.* EV-inhibitor^NC^. (F) Western blotting of the negative regulator of Stat3; SOCS3 level was increased after treatment with EV-miR-455-5p inhibitor. (G) qRT-PCR of the Stat3 regulated genes in MODE-K cells after treatment with EV-miR-455-5p inhibitor. ^**^P < 0.01 *vs.* EV, ^##^*P* < 0.01 *vs.* EV-inhibitor^NC^, n = 3. (H and I) Representative images of cell proliferation (Ki67, red) and quantitative data. EV-inhibitor^NC^ activated the proliferation of cells after radiation, while the knockdown of miR-455-5p (EV-inhibitor^455-5p^) decreased the number of Ki67^+^ cells. Scale bar = 100 μm. ^***^P < 0.001 *vs.* Ctrl, ^#^*P* < 0.05 *vs.* PBS, ^##^*P* < 0.01 *vs.* PBS, ^$^*P* < 0.05 *vs.* EV inhibitor^NC^, ^&^*P* < 0.05 *vs.* EV, n = 3. (J and K) Representative images of TUNEL staining (red) and quantitative analysis, EV-inhibitor^NC^ decreased the number of apoptotic cells following radiation injury, while the knockdown of miR-455-5p (EV-inhibitor^455-5p^) reversed the anti-apoptotic effect of EVs. Scale bar = 100 μm. ^***^P < 0.001 *vs.* Ctrl, ^###^*P* < 0.001 *vs.* PBS, ^$$$^*P* < 0.001 *vs.* EV inhibitor^NC^, ^&&&^*P* < 0.001 *vs.* EV, n = 3.

MiRNAs are a class of single-stranded small noncoding RNAs (nucleotides: approximately 20-22). They have a regulatory effect on biological cell processes 54, 55. MSC-EVs are known to mediate intercellular communications by exchanging proteins, mRNAs, and mostly miRNAs, which negatively regulate the target gene expression in different biological processes 56, 57. Then, three online miRNA databases (TargetScan, TarBase, and miRDB) were searched to obtain data that can be used to predict the candidate miRNAs of target genes. We focused on miR-455-5p (Figure 8A). In intestinal epithelial cells, miR-455-5p exerts its regulatory functions in anti-inflammation and tissue repair through the SOCS3/Stat3 signaling pathway 58, 59. MiR-455-5p downregulates SOCS3 by directly binding to the 3′ untranslated region (UTR) of SOCS3 60. The luciferase reporter assay confirmed that miR-455-5p directly interacted with SOCS3 58. To determine whether MSC-EVs transferred miR-455-5p to exert their therapeutic effect, the miR-455-5p level was evaluated. The results demonstrated that the miR-455-5p level was higher in the MSC-EVs than in the EVs from the MODE-K cells (Figure 8B). To further confirm that the MSC-EV-released miR-455-5p is shuttled to the intestine, we examined miR-455-5p expression in the intestinal tissue after the EV treatment. Figure S9A shows that the MSC-EV treatment significantly increased the miRNA-455-5p level in the intestinal tissue. Taken together, MSC-EVs attenuated the intestinal injury possibly through the miR-455-5p/Socs3/Stat3 pathway.

### Inhibition of miR-455-5p reversed the therapeutic effects of MSC-EVs on intestinal injury

**Figure 9:**
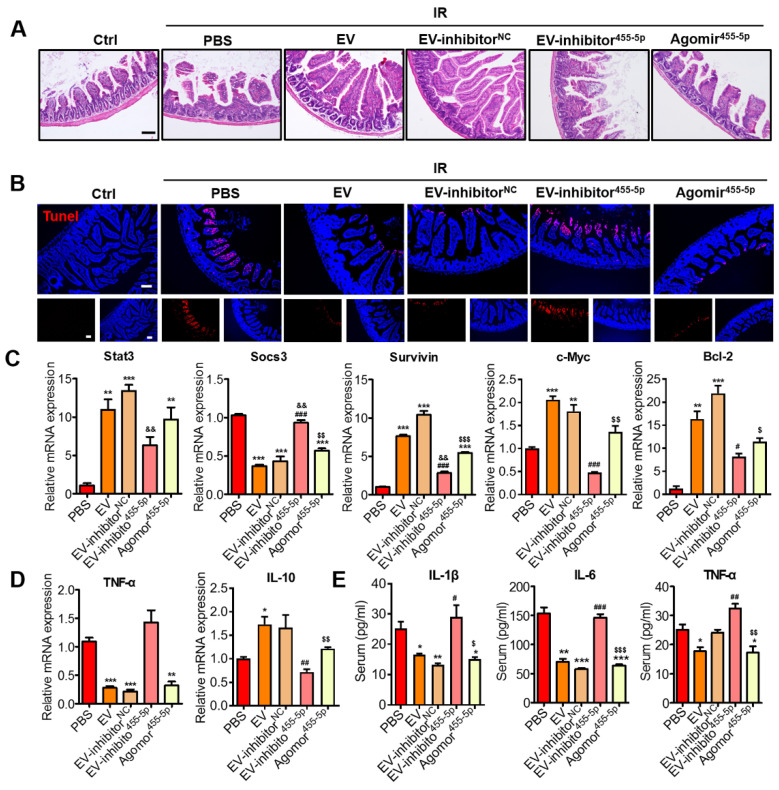
** Effects of miR-455-5p knockdown EVs on radiation-induced intestinal injury *in vivo*.** (A) Representative micrographs of H&E staining of the intestinal sections. Administration of MSC-EVs largely prevented histopathological alterations after radiation injury. MiR-455-5p agomir could improve the IR-induced intestinal injury. Scale bar = 100 μm. (B) Representative images of cell apoptosis by TUNEL staining. MSC-EVs attenuated apoptosis in the small intestines after radiation. Scale bar = 100 μm. Nuclei were counterstained with DAPI (blue). (C) qRT-PCR analysis of the intestinal tissue showed that the genes regulated by Stat3 were upregulated after treatment with MSC-EVs and miR-455-5p agomir. The negative regulator of Stat3, SOCS3 was reduced. ^**^P < 0.01 *vs.* PBS, ^***^P < 0.001 *vs.* PBS, ^#^*P* < 0.05 *vs.* EV, ^###^*P* < 0.001 *vs.* EV, ^&&^*P* < 0.01 *vs.* EV-inhibitor^NC^,^ $^*P* < 0.05 *vs.* EV-inhibitor^455-5p^, ^$$^*P* < 0.01 *vs.* EV-inhibitor^455-5p^,^ $$$^*P* < 0.001 *vs.* EV-inhibitor^455-5p^, n = 3. (D) qRT-PCR analysis of inflammation-related genes. MSC-EVs reduced the inflammatory gene expression and increased the anti-inflammatory gene expression. ^*^P < 0.05 *vs.* PBS, ^**^P < 0.01 *vs.* PBS, ^***^P < 0.001 *vs.* PBS, ^##^*P* < 0.01 *vs.* EV, ^$$^*P* < 0.01 *vs.* EV-inhibitor^455-5p^, n = 3. (E) Secretion factors in the serum were analyzed by ELISA. ^*^P < 0.05 *vs.* PBS, ^**^P < 0.01 *vs.* PBS, ^***^P < 0.001 *vs.* PBS, ^#^*P* < 0.05 *vs.* EV, ^##^*P* < 0.01 *vs.* EV, ^###^*P* < 0.001 *vs.* EV, ^$^*P* < 0.05 *vs.* EV inhibitor^455-5p^,^ $$^*P* < 0.01 *vs.* EV-inhibitor^455-5p^,^ $$$^*P* < 0.001 *vs.* EV-inhibitor^455-5p^, n = 3.

To further validate the therapeutic effect of MSC-EVs on radiation-induced intestinal injury, functional experiments were conducted *in vitro* by using the cells (Figure 8) and *in vivo* through AIR of the mice (Figure 9). To mimic radiation injury *in vitro*, the MODE-K cells were exposed to radiation and incubated with the EV inhibitor miR-455-5p or the EV inhibitor NC for 48 h (Figure 8C). A miRNA inhibitor of miR-455-5p had imperfect complementary transgenic transcripts to efficiently knockdown miR-455-5p activity, as indicated through qRT-PCR of miR-455-5p expression in EVs and the target gene SOCS3 in the MODE-K cells after treatment with the EV inhibitor miR-455-5p (Figure 8D, E). Moreover, the SOCS3 protein level remarkably increased, whereas the p-Stat3 and VEGFR1 protein level decreased in the irradiated MODE-K cells following treatment with the EV inhibitor miR-455-5p compared with the EVs and inhibitor NC (Figure 8F, Figure S9B and Figure S10). PBS and EVs served as controls. Then, cell proliferation and apoptosis were assessed. The EVs and inhibitor NC increased the expression of proliferation-related genes, Survivin and Bcl-2. By contrast, the EV inhibitor miR-455-5p attenuated the expression of these genes (Figure 8G). Ki67 staining confirmed the results (Figure 8H, I). TUNEL staining demonstrated that EV and inhibitor NC decreased the number of apoptotic cells, whereas the EV inhibitor miR-455-5p reversed the anti-apoptotic effect of EVs (Figure 8J, K).

### MiR-455-5p agomir further verified the miR-455-5p role of MSC-EVs in irradiation-induced intestinal injury *in vivo*

Using the miR-455-5p agomir through the AIR mouse model, we examined the effects of miR-455-5p on the regulation of antiapoptosis and anti-inflammation. After exposure to 15-Gy AIR (or not irradiated as controls), the mice were randomized into six groups: non-irradiation group (control) and irradiation groups.

The irradiation groups included the PBS, EV, EV miR-455-5p inhibitor NC, EV miR-455-5p inhibitor, and miR-455-5p agomir groups. From the day of irradiation, during the next 3 days, the mice were intravenously injected with PBS (control) or 100 μg (in 100 μL PBS) of EVs or EVs isolated from the miR-455-5p inhibitor- or miR-455-5p agomir-treated MSCs. On day 4, the intestinal tissues and serum specimens were collected for immunohistochemistry and inflammatory factor analysis. According to the H&E staining results, miR-455-5p agomir improved the irradiation-induced intestinal injury, which was indicated by an increased number of crypts and integrity of the villi (Figure 9A). TUNEL-positive cells decreased remarkably in the miR-455-5p agomir treatment compared with the EV miR-455-5p inhibitor group (Figure 9B). Moreover, the expression of antiapoptotic (Bcl-2) and pro-proliferative (Survivin and C-Myc) genes present downstream of the Stat3 signaling pathway were examined and found to mediate injury healing. Compared with the EV miR-455-5p inhibitor group, the miR-455-5p agomir group exhibited a significant increase in Stat3, Survivin, C-Myc, and Bcl-2 expression, but it decreased SOCS3 expression in the AIR mouse (Figure 9C).

To investigate whether miR-455-5p agomir modulated the inflammatory response in the AIR mouse, the levels of inflammatory cytokines in each group were determined using qRT-PCR and ELISA. Unlike in the IR+PBS and IR+EV inhibitor miR-455-5p groups, which exhibited elevated mRNA levels of the pro-inflammatory cytokine TNF-α, treatment of the mouse with EVs, EV miR-455-5p inhibitor NC, and miR-455-5p agomir significantly reduced the expression of this cytokine but augmented anti-inflammatory IL-10 expression (Figure 9D). Consistent with this result, the production of IL-1β, IL-6, and TNF-α decreased in the serum from the EV- and miR-455-5p agomir-treated AIR mice, compared with the PBS- and EV miR-455-5p inhibitor-treated mice (Figure 9E). These results indicated that miR-455-5p agomir reduced intestinal inflammation in the AIR mouse.

Overall, miR-455-5p-containing EVs reduced SOCS3 levels and activated Stat3 levels. Hence, they reduced cell death and enhanced cell survival, which explains the therapeutic effects of MSC-EVs on intestinal injury.

## Discussion

Radiation-induced intestinal injury is a crucial cause of acute GIT symptoms and death in patients receiving radiotherapy for pelvic and abdominal tumors. To date, no effective drugs are available for preventing intestinal injury in the clinic. Developing protective or therapeutic agents against radiation-induced damage of gastrointestinal cells is critical 2. MSC therapy has been used to treat human diseases in numerous experimental and clinical studies 61, as well as to treat radiation damage 62. Evidence that the production and release of secretory factors from MSCs mediate the therapeutic effects of these cells is growing; these factors are referred to as secretomes 63. Because EVs are a part of MSC secretomes, EVs released by MSCs are reported to be closely related to the MSC regenerative properties 64. Current evidence suggests that MSC-EVs have a greater therapeutic potential than MSCs. Compared with MSCs, MSC-EVs carry many anti-inflammatory agents, such as miRNAs, mRNAs, and proteins. These agents have low immunogenicity and can be easily stored and administered for therapeutic purposes 18, 64-66. MSC-EVs are very promising in nanomedicine because of their beneficial biological properties and excellent therapeutic potential against inflammatory 67 and degenerative diseases 68, 69. MSC-EVs alleviated experimental colitis in mice by inhibiting inflammation and oxidative stress 70. MSC-EVs were effective against radiation-induced enteritis and essential for the proliferation and differentiation of Lgr5^+^ intestinal epithelial stem cells by regulating the mir-195/Akt/β-catenin pathway 71. They also promoted epithelial repair and intestinal epithelium regeneration in mice with radiation-induced gastrointestinal toxicity 72. However, there were few studies has reported the radiation damage tropism of MSC-EVs and the specific molecular mechanism through which a specific component present in them exerts a damage-repair effect on radiation-induced intestinal injury.

In this study, the fate and biodistribution of MSC-EVs was first investigated *in vivo*. The MSC-EVs were labeled with the ExoGlow-*Vivo* dye, a non-lipophilic dye that emits in the NIR range. The labeled MSC-EVs were intravenously injected into mice to visualize the biodistribution and trend of MSC-EVs by using an IVIS *in vivo* imaging system. According to molecular imaging, the MSC-EVs exhibited preferable selectivity to radiation-injured intestines and can be used for real-time monitoring of the severity of radiation intestinal injury at the early stage.

MSC-EVs are key players in wound healing and tissue regeneration 12 and can aggregate to the injury site like MSCs. However, the mechanism through which MSC-EVs target damaged sites remains unclear. MFGE8, as a lipophilic glycoprotein on the cell membrane, may participate in various cell-to-cell interactions and has multiple functions, such as anti-inflammation, and promotion of macrophage phagocytosis of apoptotic cells, angiogenesis, and mucosal repair 73. MFGE8 is enriched in MSC-EVs and is located on the outside of the EVs 74. It can bind to PS on apoptotic cells 3. Treatment with the exogenous recombinant MFG-E8 in a cecal ligation and puncture-induced mouse sepsis model accelerated intestinal mucosa healing by binding to exposed PS receptors on the injured intestinal epithelial cells 75. According to our results, MSC-EVs targeted the injured intestine by mediating the binding of MFGE8 to PS on the injured intestinal epithelial cells and releasing therapeutic factors, thereby protecting the MODE-K cells or mice against radiation-induced injury (Scheme 1).

Furthermore, we explored the connection between “MSC-EV-mediated binding of MFGE8 to PS” and “Stat3 pathway” by using the MFGE8-neutralizing antibody and annexin V to neutralize the MFGE8 protein on the MSC-EVs and block the PS on the irradiated MODE-K cell membranes, respectively. The expression of Stat3 pathway-related proteins was downregulated after irradiation. The protein expression was significantly higher in the MSC-EV-treated group than in the irradiation group, whereas the protein expression in the Annexin V treated after radiation and then co-cultured with MSC-EVs pre-treated with MFGE8 neutralizing antibody group decreased (Figure S8C). In summary, MSC-EVs targeted the irradiated intestine through binding of MFGE8 to PS on irradiated cells and releasing therapeutic factors that exert therapeutic effects.

Moreover, we investigated the therapeutic effects and revealed the mechanisms underlying the action of MSC-EVs in radiation-induced intestinal injury. After irradiation, the MSC-EV treatment protected the MODE-K cells or mice against radiation-induced injury, including cell death (Figure 4) and DNA damage (Figure S4); improved cell proliferation capacities (Figure 4); and alleviated cellular and histological responses to radiation (Figure 5). Furthermore, MSC-EVs were found to repair radiation-induced intestinal injury by regulating the Stat3 signaling pathway (Figure 7). This suggested that MSC-EVs can be a potential nanotherapeutic agent for radiation injury treatment.

Furthermore, an attractive feature of using MSC-EVs as a targeted therapeutic agent for intestinal injury is the naturally beneficial components contained in them. MSC-EVs have a complex mixture of RNAs and proteins targeting different pathways, which enable EVs to exert their therapeutic efficacy. Being a major part of the EV cargo, miRNAs are known to play a vital role in mediating EV function. MiRNAs are pivotal players in mediating exocrine function 76. Because radiation-induced intestinal injury involves multiple interactions among injured intestines, epithelial cells, phagocytic cells, and secretory cells, it is highly regulated by complicated signaling networks. This indicates that targeting a certain cell type to regulate a single pathogenic process might be insufficient for protecting against radiation-induced intestinal injury. Therefore, holistic integrative therapy, such as EVs carrying various therapeutic molecules, is the preferred therapeutic solution against radiation-induced injuries. Although the present study focused on miR-455-5p in MSC-EVs, other components might also contribute partially to the beneficial therapeutic effects against radiation injury. In our future work, we will explore the therapeutic mechanisms of other miRNAs or effective factors 77 in MSC-EVs to treat radiation-induced intestinal injury.

Stats mediate cytokine signaling. Constitutively activated Stats, especially Stat3, is involved in several inflammatory and malignant disorders. Stat3 activation in the intestinal epithelial cells of animal models is vital for acute wound healing responses 59. We here assessed the expression of Stat3 and its downstream negative regulator target gene SOCS3. Consistent with previous reports 58, our results unveiled that MSC-EVs remarkably decreased SOCS3 levels and in turn activated Stat3 signaling, including its downstream genes, such as Survivin, c-Myc, and Bcl-2 (Figure 7). The MSC-EVs improved viability, reduced radiation-induced apoptosis and inflammation of intestinal epithelial cells, and ameliorated radiation-induced intestinal damage.

Beyond the therapeutic functions, noninvasively injected MSC-EVs also exhibited an impressive diagnostic potential in the radiation-induced intestinal injury model. The MSC-EVs demonstrated excellent irradiated intestine-targeted properties, extended retention time, and outstanding therapeutic effects, which indicated that they represent a viable treatment approach for radiation-induced intestinal injury. However, the radiation injury-targeted mechanism and binding molecules remain largely unclear. According to some studies, MFGE8 has a high binding activity toward anionic phospholipids, especially PS 78. MFGE8 thus specifically binds the lipid bilayer-exposing PS such as that present on the membrane of apoptotic cells 79. Moreover, following after myocardial ischemic injury, MSC-EVs served as an opsonin for apoptotic cells and activated the macrophage phagocytosis ability through MFGE8 3. In this study, MFGE8 in the MSC-EVs played an essential role in targeting radiation-induced intestinal injury via PS present in the irradiated intestinal cells. Therefore, MSC-EVs are promising as dependable molecular therapeutics for the diagnosis and personalized medicine of radiation-induced injury. This advance offers a new and compelling perspective on the field of MSC-EV-based therapy and an innovative framework for addressing radiation injury-posed challenges.

## Conclusions

In conclusion, the natural, nontoxic MSC-EVs preferentially targeted the irradiated intestine and possessed anti-inflammatory and pro-repair functions. These results suggest MSC-EVs can be used as a nanotherapeutic personalized medicine for radiation-induced intestinal injury, which will allow the clinical transformation of MSC-EV-based therapy.

## Supplementary Material

Supplementary figures and table.

## Abbreviations

MSC: Mesenchymal stem cells
EVs: Extracellular vesicles
IR: Ionizing radiation
MFGE8: Milk fat globule EGF factor 8
AIR: Abdominal irradiation
Stat3: Signal transducers and activators of transcription 3
IL: Interleukin
FBS: Fetal bovine serum
DMEM: Dulbecco's modified Eagle's medium
TNF-α: Tumor necrosis factor α
