# Supplementary material for: Mesenchymal stem cell-derived extracellular vesicles targeting irradiated intestine exert therapeutic effects
Source: Theranostics. 2024 Aug 26;14(14):5492–511. doi: 10.7150/thno.97623 (PMC11413785; doi:10.7150/thno.97623)
Supplement: Supplementary file 1 — Supplementary figures and table. [file thnov14p5492s1.pdf]

Supporting Information

Mesenchymal stem cell-derived extracellular vesicles targeting irradiated intestine exert therapeutic effects

Ningning He[1#], Mingxin Dong[2#], Yuxiao Sun[1#], Mengmeng Yang[1],Yan Wang[1], Liqing Du[1], Kaihua Ji[1], Jinhan Wang[1], Manman Zhang[1], Yeqing Gu[1], Xinran Lu[1], Yang Liu[1], Qin Wang[1], Zongjin Li[3], Huijuan Song[1*], Chang Xu[1*] and Qiang Liu[1*]

[1]Tianjin Key Laboratory of Radiation Medicine and Molecular Nuclear Medicine, Institute of Radiation Medicine, State Key Laboratory of Advanced Medical Materials and Devices, Chinese Academy of Medical Science and Peking Union Medical College, Tianjin 300192, China.

[2]State Key Laboratory of Experimental Hematology, National Clinical Research Center for Blood Diseases, Haihe Laboratory of Cell Ecosystem, Institute of Hematology & Blood Diseases Hospital, Chinese Academy of Medical Sciences & Peking Union Medical College, Tianjin, 300020, China.

[3]School of Medicine, Nankai University, Tianjin, China.

[#]These authors contributed equally to this work.

[*]Correspondence: Qiang Liu，Institute of Radiation Medicine, Chinese Academy of Medical Sciences & Peking Union Medical College, Tianjin Key Laboratory of Molecular Nuclear Medicine, Tianjin, 300192, China. Ph:+86-22-8568-3008, Fax: +86-22-8568-3033, E-mail: liuqiang@irm-cams.ac.cn; Chang Xu, Institute of Radiation Medicine, Chinese Academy of Medical Sciences & Peking Union Medical College, Tianjin Key Laboratory of Molecular Nuclear Medicine, Tianjin, 300192, China. E-mail: xuchang@irm-cams.ac.cn; Huijuan Song,

Institute of Radiation Medicine, Chinese Academy of Medical Sciences & Peking Union Medical College, Tianjin Key Laboratory of Molecular Nuclear Medicine, Tianjin, 300192, China. E-mail: songhuijuan@irm-cams.ac.cn.

Supporting Information Contents:

Table S1

Figures S1-S10

Table S1. Primer sequences used for real-time RT-PCR.

Gene	Forward primer (5'→3')	Reverse primer (5'→3')
GADPH	AGGTCGGTGTGAACGGATTTG	TGTAGACCATGTAGTTGAGGTCA
IL-1β	GCAACTGTTCCTGAACTCAACT	ATCTTTTGGGGTCCGTCAACT
IL-6	TAGTCCTTCCTACCCCAATTTCC	TTGGTCCTTAGCCACTCCTTC
TNF-α	CCTGTAGCCCACGTCGTAG	GGGAGTAGACAAGGTACAACCC
IL-10	GCTCTTACTGACTGGCATGAG	CGCAGCTCTAGGAGCATGTG
IL-22	AATGAAAAGGCCCCCAAGGTAGTTATCC	GTCGTTTCCGCAACAAGTCCTCTTC
Stat3	GCCATCCTAAGCACAAAGCC	GGGAATGTCGGGGTAGAGGT
c-Myc	GACTGTATGTGGAGCGGTTTCT	TGCTGTCGTTGAGCGGGTA
Survivin	ATCGCCACCTTCAAGAACTG	AATCAGGCTCGTTCTCGGTA
VEGF	AACGATGAAGCCCTGGAGTG	TGAGAGGTCTGGTTCCCGA
Bcl-2	GATGACTGAGTACCTGAACCG	CAGAGACAGCCAGGAGAAATC
SOCS3	GGAGAGCGGATTCTACTGGA	TGACGCTCAACGTGAAGAAG
Reg3b	ACTCCCTGAAGAATATACCCTCC	CGCTATTGAGCACAGATACGAG

Reg3g	ATGCTTCCCCGTATAACCATCA	GGCCATATCTGCATCATACCAG

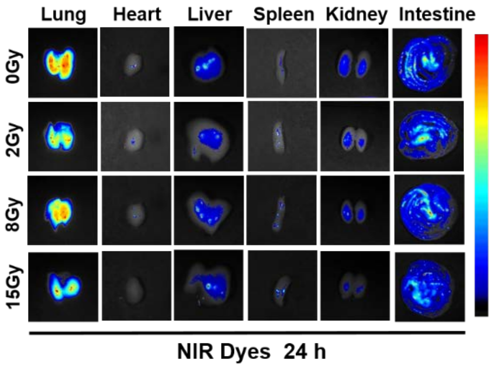

Figure S1. *Ex vivo* imaging of NIR dyes in radiation-induced intestinal injury mice. There showed no radiation dose-dependent of NIR dyes distribution in intestines.

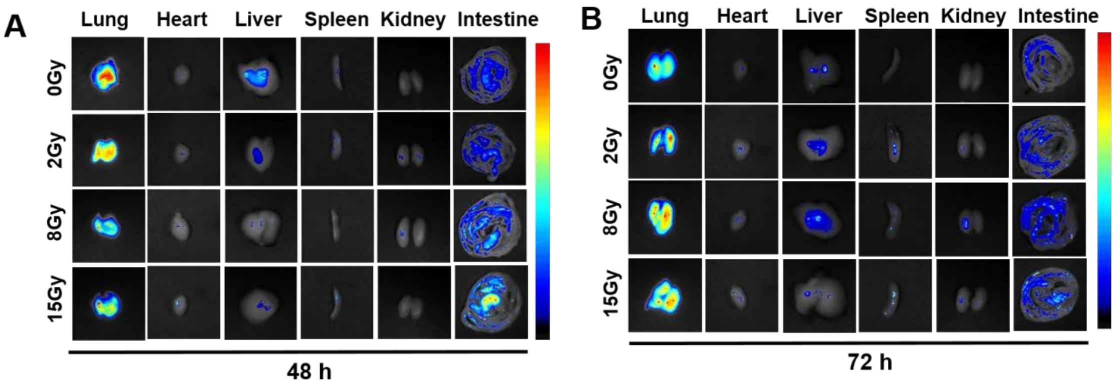

Figure S2. *Ex vivo* imaging of MSC-EVs in radiation-induced intestinal injury mice. (A) Distributions of NIR-labeled EVs at 48 h after NIR-EVs administration. (B) Distributions of NIR-labeled EVs at 72 h after NIR-EVs administration.

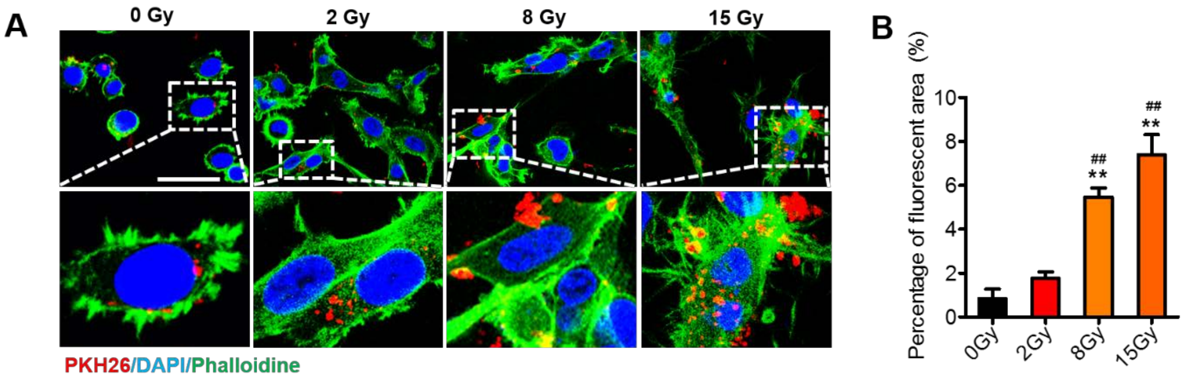

Figure S3. Radiation increase cellular uptake of MSC-EVs. (A) Representative immunofluorescence images showing uptake of PKH26-labeled MSC-EVs by MODE-K cells under different irradiation doses. Scale bar, 25 μm. (B) Graph showing the percentage uptake of fluorescent MSC-EVs (red) by MODE-K cells. The results showing radiation increases the binding of MSC-EVs to irradiated MODE-K cells. $^{**}P < 0.01$ *vs.* 0 Gy, $^{\#\#}P < 0.01$ *vs.* 2 Gy, n=3.

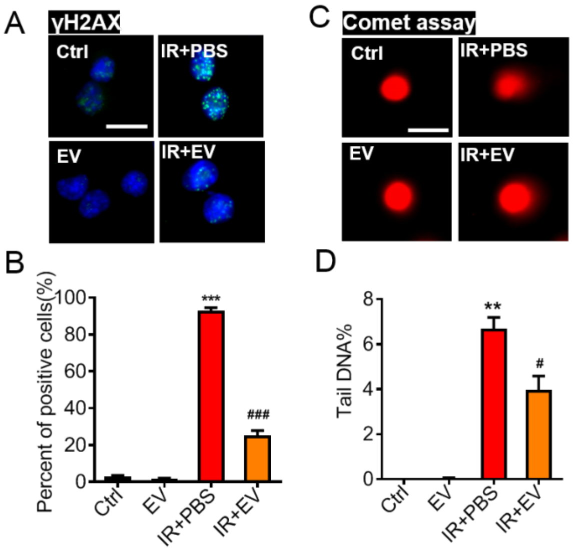

Figure S4. Effects of MSC-EVs on DNA-damage Responses. (A and B) Representative images of γH2AX (green) and quantitative data. Scale bar, 100 μm. (C and D) Representative images of comet assay and quantitative data. MSC-EVs ameliorated DNA damage after radiation. Scale bar, 100 μm. $^{**}P < 0.01$ *vs.* EV, $^{***}P < 0.001$ *vs.* EV, $^{\#}P < 0.05$ *vs.* IR+PBS, $^{\#\#\#}P < 0.001$ *vs.* IR+PBS, n=3.

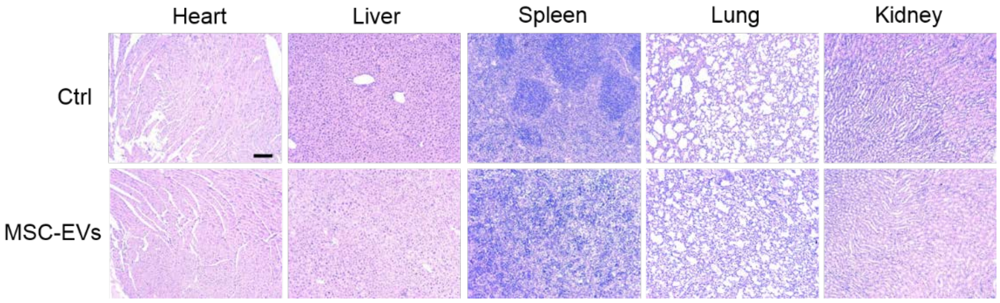

Figure S5. Micrographs for H&E staining of organs sections. There was no difference between the MSC-EVs administration group and the control group. Scale bar, 100 μm.

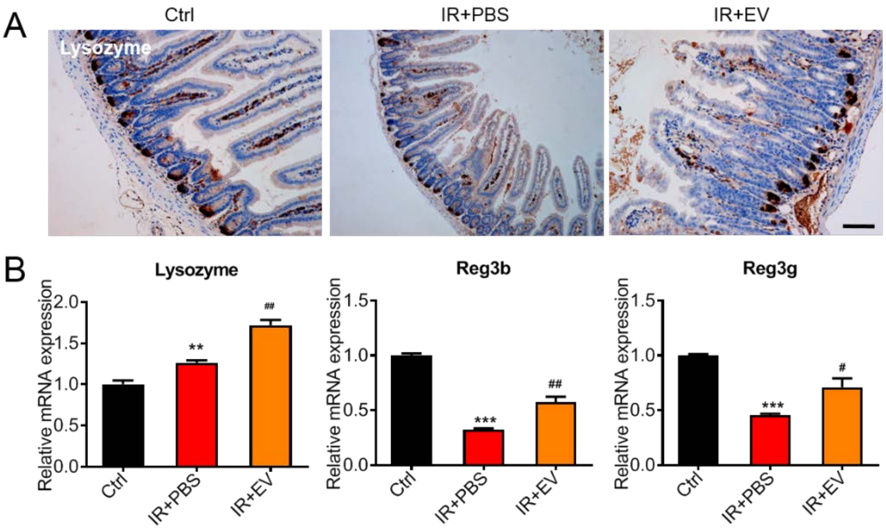

Figure S6. The effect of MSC-EVs on the protection of intestinal cells in irradiated mice. (A) Immunohistochemistry of Lysozyme in intestine tissues. Scale bar, 100 μm. (B) qRT-PCR of Lysozyme, and the expressions of regeneration-associated genes (Reg3b and Reg3g) were increased in the MSC-EV group. ***P* < 0.01 *vs.* Ctrl, ****P* < 0.001 *vs.* Ctrl, $^{\#}P < 0.05$ *vs.* IR+PBS, $^{\#\#}P < 0.01$ *vs.* IR+PBS, n=3.

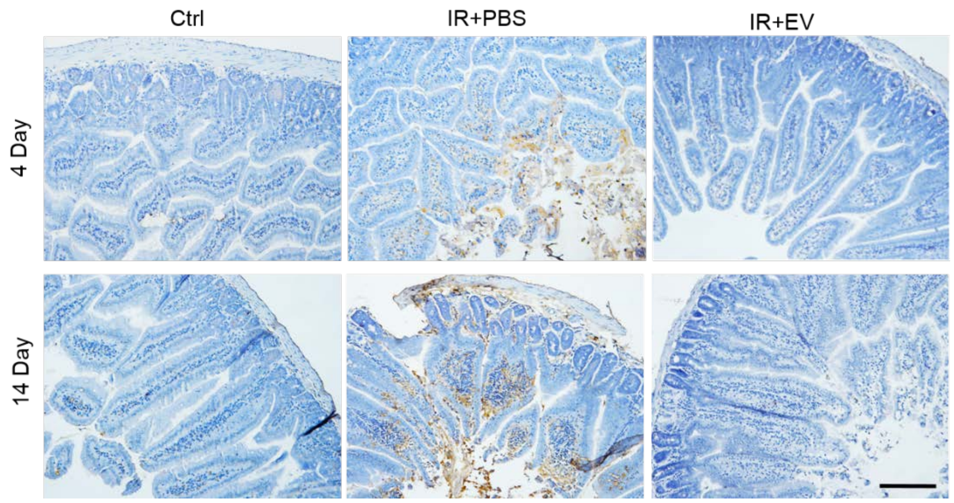

Figure S7. Immunohistochemistry of TUNEL in intestine tissues. MSC-EVs significantly reduced the apoptosis level in the intestine of mice after radiation.

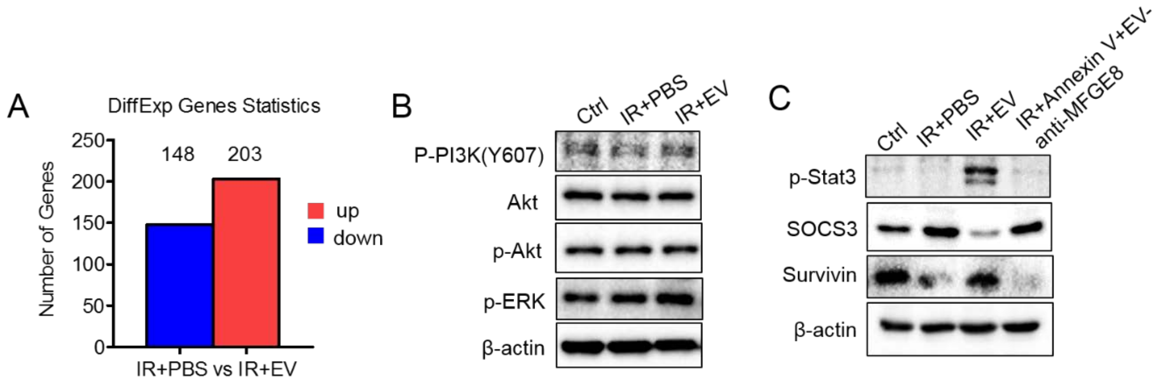

Figure S8. (A) Statistical histogram of differential genes. Red represents up-regulated genes, blue represents down-regulated genes, and the ordinate represents the number of differential genes. (B) Western blot of Akt and ERK pathway related protein expression. There was no obvious difference among three groups. (C) Western blot of Stat3 pathway related protein expression. The Stat3 pathway was inactivated after blocked the PS on cell membrane and neutralized the MFGE8 protein on the MSC-EVs.

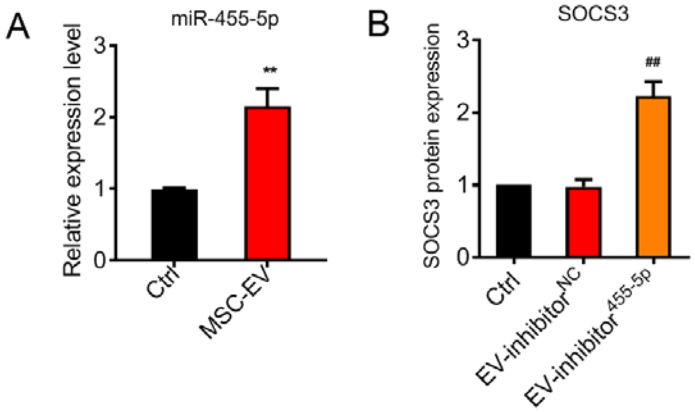

Figure S9. (A) qRT-PCR analysis of the intestinal tissue showed that MSC-EVs treatment significantly increased the level of miRNA-455-5p in the intestinal tissue. $^{**}P < 0.01$ *vs.* Ctrl, n=3. (B) Quantification analysis of the SOCS3 protein in Figure 8F. $^{\#\#}P < 0.01$ *vs.* EV-inhibitor^{NC}, n=3.

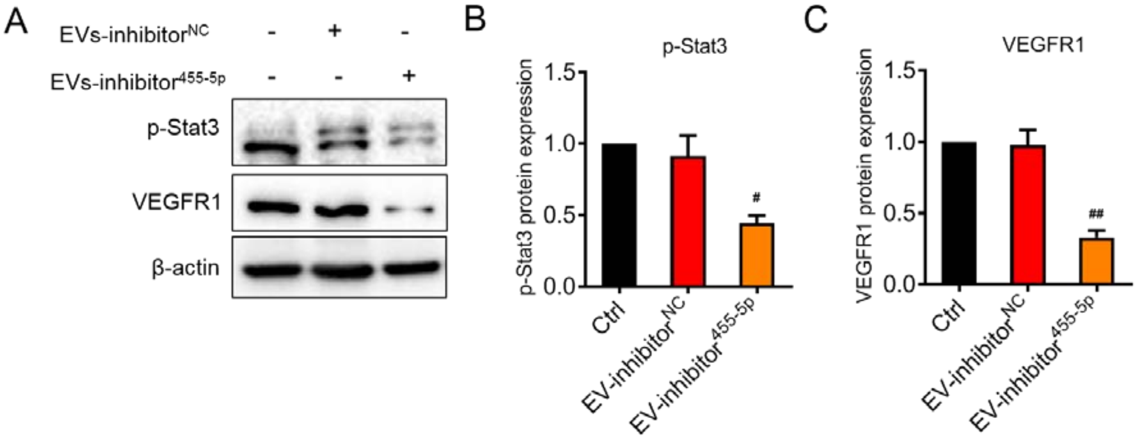

Figure S10. (A) Western blot of Stat3 pathway related protein expression. p-Stat3 and VEGFR1 protein level were inhibited after treatment with EV-miR-455-5p inhibitor. $^{\#}P < 0.05$ *vs.* EV- inhibitor^{NC}, $^{\#\#}P < 0.01$ *vs.* EV-inhibitor^{NC}, n=3.
